# DMSO cryopreservation is the method of choice to preserve cells for droplet-based single-cell RNA sequencing

**DOI:** 10.1038/s41598-019-46932-z

**Published:** 2019-07-23

**Authors:** Christian T. Wohnhaas, Germán G. Leparc, Francesc Fernandez-Albert, David Kind, Florian Gantner, Coralie Viollet, Tobias Hildebrandt, Patrick Baum

**Affiliations:** 1 Computational Biology, Boehringer Ingelheim Pharma GmbH & Co. KG, 88397 Biberach, Germany; 2 Department of Biology, University of Konstanz, 78457 Konstanz, Germany; 3 Translational Medicine & Clinical Pharmacology, Boehringer Ingelheim Pharma GmbH & Co. KG, 88397 Biberach, Germany

**Keywords:** Transcriptomics, Transcriptomics

## Abstract

Combining single-cell RNA sequencing (scRNA-seq) with upstream cell preservation procedures such as cryopreservation or methanol fixation has recently become more common. By separating cell handling and preparation, from downstream library generation, scRNA-seq workflows are more flexible and manageable. However, the inherent transcriptomic changes associated with cell preservation and how they may bias further downstream analysis remain unknown. Here, we present a side-by-side droplet-based scRNA-seq analysis, comparing the gold standard – fresh cells – to three different cell preservation workflows: dimethyl sulfoxide based cryopreservation, methanol fixation and CellCover reagent. Cryopreservation proved to be the most robust protocol, maximizing both cell integrity and low background ambient RNA. Importantly, gene expression profiles from fresh cells correlated most with those of cryopreserved cells. Such similarities were consistently observed across the tested cell lines (R ≥ 0.97), monocyte-derived macrophages (R = 0.97) and immune cells (R = 0.99). In contrast, both methanol fixation and CellCover preservation showed an increased ambient RNA background and an overall lower gene expression correlation to fresh cells. Thus, our results demonstrate the superiority of cryopreservation over other cell preservation methods. We expect our comparative study to provide single-cell omics researchers invaluable support when integrating cell preservation into their scRNA-seq studies.

## Introduction

Research at the single-cell level has become one of the most rapidly growing disciplines in life science, allowing for new insights into the dynamics of biological processes and tissue composition^1^. In genomics, single-cell RNA sequencing (scRNA-seq) has substantially driven this development by shifting transcriptomic analysis from population scale to single-cell resolution. This unbiased, next-generation sequencing based approach increases the resolution of gene expression analyses and allows comprehensive investigation of complex biological systems such as tissues or the immune system and the discovery of unknown cell subsets^2,3^.

Since its first description in 2009^4^, further development has led to an explosion of scRNA-seq systems^3^, including platforms that differ in the way cells are isolated, how transcripts are captured and amplified as well as their sensitivity and throughput^3^. High-throughput droplet-based platforms such as inDrop^5^, Drop-seq^6^ and the commercial Chromium^7^ system apply microfluidics to encapsulate single cells into droplets. Distinctly barcoded beads carrying cell barcodes and unique molecular identifiers (UMIs) are co-encapsulated into the droplets in order to individually tag the captured transcripts per cell. This droplet approach enables the investigation of tens of thousands of individual cells simultaneously in a single run^2,3^. The ability to process large amounts of cells per sample facilitates the detection of rare cell types and is one of the main advantages of droplet-based systems^2^ which has contributed greatly to the popularity of these techniques. Although droplet-based platforms allow for a high throughput of cells per sample, the capacity to process different samples in parallel is limited. Reasons for limited sample throughput are multiple, such as restrictions in parallelised sample processing by microfluidic devices or the requirement to prepare single-cell suspensions of high-quality for encapsulation which is essential for a successful scRNA-seq study^1^. Furthermore, the preparation of high-quality cell suspensions can be very time-consuming by itself, thereby limiting sample throughput. Possibilities to separate sample preparation from immediate processing in the microfluidic device would therefore allow for greater throughput and flexibility. Additionally, immediate processing of samples can be a challenge due to a lack of dedicated equipment, such as microfluidic devices, and infrastructure^1^. Moreover, simultaneous instead of immediate sample processing might be the preferred choice if samples were collected across several time points, such as time course analysis, to prevent technical batch effects^1^. Similarly, studies with spatially separated cell handling and scRNA-seq analysis, such as multicentered trials, require methods to separate cell preparation from immediate processing for scRNA-seq.

Recently, some efforts have been made to develop protocols that enable storage and preservation of cells for later scRNA-seq analysis^7–13^. Most popular are protocols where cells are cryopreserved using dimethyl sulfoxide (DMSO) or preserved by methanol fixation. DMSO, a cell penetrating cryoprotectant^14^, is commonly used to cryopreserve animal cells and protects them from intracellular formation of ice crystals^15^. Methanol, in contrast, is a coagulating fixative which dehydrates cells and cause nucleic acids to appear in a collapsed form at concentrations >65% and in the presence of salts^8^. This procedure allows the preservation of nucleic acids while introducing only minor chemical modifications^16^. Nucleic acids can then be transformed into their original state by rehydration^8^ for further processing. Compatibility of preservation by 80% methanol has previously been shown for bulk and scRNA-seq^8,17^. Commercially available formulations to preserve cells and their RNAs provide an additional source of reagents for scRNA-seq-compatible cell preservation. One such reagent is CellCover (Anacyte Laboratories, Hamburg, Germany) that allows preservation of distinct cell types and their RNAs as well as proteins. The CellCover reagent has already been successfully applied to stabilize cells during cell preparation until processing them for scRNA-seq^18^.

Although these methods were used to preserve or stabilize cells for scRNA-seq in different studies^7,8,12,19^, a systematic comparison is still lacking. In this study, we compared DMSO cryopreservation, methanol fixation as well as CellCover reagent to preserve cells for scRNA-seq analysis using droplet-based high-throughput platforms. These three preservation methods were compared using a species mixing experiment with human (HEK293) and murine (3T3) cell lines, as well as monocyte-derived macrophages (MDMs) which represent a more difficult to preserve primary cell derived cell type. Protocol assessment was based on quality control parameters including cell integrity, gene and UMI count per cell, percentage of mitochondrial transcripts per cell and cross-species contamination. Additionally, we investigated whether cell preservation affects the gene expression profiles. Finally, the best method was tested with immune cells in order to assess its performance on a heterogeneous population of primary cells that are commonly investigated in scRNA-seq studies.

## Methods

### Ethics statement

All studies on human donor blood were performed in accordance with the guidelines and regulations of German legislation and the experimental protocol was approved by the ethical committee of the Landesärztekammer Baden-Württemberg (Germany). For this study an anonymized blood sample was obtained from a healthy volunteer that provided written informed consent. Animal experiments were conducted in accordance with the German law on animal welfare (TierSchG) and were approved by the Regierungspräsidium Tübingen. All methods were performed in accordance with relevant guidelines and applicable regulations.

### Samples and cell preparation for scRNA-seq

#### Species mixing experiment

Human HEK293 and murine NIH 3T3 cells were grown in Dulbecco’s Modified Eagle Medium (DMEM, Gibco, Thermo Fisher Scientific, Waltham, MA) supplemented with 10% of fetal calf serum (FCS, Gibco, Thermo Fisher Scientific, Waltham, MA). The cells were incubated at 37 °C and 5% CO_2_ to approximately 70–80% confluence and then prepared for scRNA-seq analysis and preservation.

First, the medium was removed and the cells were washed twice with 10 mL of PBS (Gibco, Thermo Fisher Scientific, Waltham, MA). Cells were detached using 3 mL of Trypsin-EDTA solution (Sigma-Aldrich, St. Louis, MO) and incubation for 30 sec at 37 °C. Trypsinization was stopped by adding 10 mL of medium and cells of several flasks were collected and centrifuged for 5 min at 300 g. The supernatant was removed and the cells were resuspended in 5 mL of PBS/bovine serum albumin (BSA) buffer (0.01% BSA, Sigma-Aldrich, St. Louis, MO). The cells were then stored on ice and filtered through a 40 µm cell strainer (Corning, New York, NY) for scRNA-seq and preservation by the different preservation methods.

#### Monocyte-derived macrophages

Monocyte-derived macrophages were differentiated from peripheral blood monocytes (Supplementary Methods) and collected for scRNA-seq analysis by incubating UpCell^TM^ plates (Thermo Fisher Scientific, Waltham, MA) for approximately 30 min at room temperature until the cells detached from the plates. MDMs were then pelleted for 10 min at 300 g, resuspendend in PBS/2 mM EDTA/2% FCS and stored on ice for 15 min. For scRNA-seq and cell preservation the MDMs were resuspended in PBS/BSA buffer and filtered through a 40 µm cell strainer.

#### Rat liver immune cells

Immune cells were isolated from livers of male Han Wistar rats (Janvier, Le Genest-St-Isle, France) fed with either choline-deficient, l-amino acid-defined (CDAA) diet (n = 2, positive control (PC)) or choline-sufficient, l-amino acid-defined (CSAA) diet (n = 2, negative control (NC)). Livers were dissected, pushed through 500 µm and 200 µm filters, respectively and then flushed through a 70 µm cell strainer (pluriSelect, Leipzig, Germany) using PBS/0.5% FCS buffer. In between the different filtering steps the liver suspensions were centrifuged for 5 min at 320 g and 4 °C. Cell pellets were washed again using chilled PBS/0.5% FCS buffer and the remaining erythrocytes were lysed using 12 mL of RBC Lysis Buffer (Invitrogen, Thermo Fisher Scientific, Waltham, MA) according to the manufacturer´s instructions. The cell pellet was washed and resuspended in 15 mL of PBS/0.5% FCS buffer and carefully transferred on top of 12 mL Lymphocyte Separation Medium (Lonza, Basel, Switzerland). Centrifugation for 20 min at 400 g and 4 °C (brake set to 7, centrifuge model 5810 R, Eppendorf, Hamburg, Germany) separated the different cell populations and the immune cell containing gradient interphase was collected and washed in PBS/0.5% FCS buffer. To prepare cells for scRNA-seq the cell pellets were washed in 10 mL of HBSS/0.04% BSA/2 mM EDTA and HBSS/0.04% BSA, respectively. Finally, the cells were resuspended in HBSS/0.04% BSA buffer and filtered through a 40 µm cell strainer.

#### Estimation of cell concentration and integrity

Cell concentration and cell integrity/viability were determined by trypan blue dye exclusion staining and the Countess^TM^ cell counter (Invitrogen, Thermo Fisher Scientific, Waltham, MA) for the species mixing experiment and MDMs. For rat liver immune cells both parameters were estimated by the NucleoCounter NC-200^TM^ device (Chemometec, Allerod, Denmark; acridine orange and DAPI staining).

### Cell preservation and processing for scRNA-seq

Three methods to preserve cells for later scRNA-seq analysis were evaluated in this study, cryopreservation using DMSO, methanol fixation and storage in CellCover reagent (Anacyte Laboratories, Hamburg, Germany) at −20 °C and 4 °C. All methods were tested side-by-side for the species mixing experiment and MDMs whereas only DMSO cryopreservation was tested for the rat immune cells. Cells of the species mixing experiment were stored for one and 15 weeks (except the preservation by CellCover reagent at 4 °C which was only tested for one week), respectively to investigate the effect of storage duration. MDMs and rat immune cells were stored for three weeks.

#### DMSO cryopreservation

Cells were cryopreserved using a modified version of the protocol described by 10x Genomics (CG00039, Rev C). Briefly, cell suspensions were centrifuged for 5 min at 300 g and 4 °C. Supernatants were discarded and the cell pellets were resuspended in pre-chilled (4 °C) DMSO/FCS solution containing 10% of DMSO. Aliquots of 1 mL were dispensed into cryovials and placed into a CoolCell^®^ (BioCision, Larkspur, CA) that was pre-cooled in the fridge at 4 °C for at least two hours. The CoolCell^®^ was stored overnight at −80 °C and the cryovials were then transferred to −150 °C storage. Aliquots contained 4.5 × 10^6^, 3.2 × 10^6^ and between 2.1 × 10^6^ and 8.5 × 10^4^ cells for the species mixing experiment, MDMs and rat liver immune cells, respectively.

Frozen samples were prepared for scRNA-seq by rapidly thawing them at 37 °C and transferring the cells into 50 mL centrifuge tubes. The cryovials were rinsed with 1 mL of warm (37 °C) medium which was then added dropwise to the DMSO containing fraction while gently shaking the cells. DMEM was used for the species mixing experiment while RPMI 1640 medium (Gibco, Thermo Fisher Scientific, Waltham, MA) supplemented with 10% of FCS was used for primary and primary-derived cells. Next, the cells were gradually diluted by first adding 2 mL of medium followed by another 4, 8 and 16 mL respectively. The cell suspension was gently swirled for 5 sec and incubated for 1 min in between the four dilution steps. The diluted cell suspension was centrifuged for 5 min at 300 g and most of the medium was discarded leaving approximately 1 mL of supernatant. The cells were gently resuspended in the remaining supernatant and washed by adding 9 mL of medium. After centrifugation for 5 min at 300 g the supernatant was discarded and the cells were washed in 1.5 mL of PBS/BSA buffer. Finally, the cells were resuspended in PBS/BSA buffer, filtered through a 40 µm cell strainer and stored on ice until counting and loading of the cells into the scRNA-seq device. For immune cells HBSS was used instead of PBS.

#### Methanol fixation

Methanol fixation of species mixing cell lines and MDMs was performed as described by Alles *et al*.^8^. Briefly, cells were pelleted by centrifugation for 5 min at 300 g and 4 °C and resuspended in ice-cold PBS/BSA. Methanol (JT Baker, Avantor Center Valley, PA) pre-chilled to −20 °C was added drop-wise to finally obtain a methanol concentration of 80%. The cells were gently mixed while adding methanol to avoid formation of cell clumps. 1 mL aliquots containing 4.5 × 10^6^ HEK/3T3 cells or 3.2 × 10^6^ MDMs were stored on ice for 20 min and then transferred to −80 °C. For scRNA-seq analysis the preserved cells were processed as described before^8^.

#### CellCover reagent

In order to preserve cells using the CellCover reagent, HEK/3T3 cells and MDMs were centrifuged for 5 min at 300 g and 4 °C. The supernatant was removed and cell pellets were resuspended in cold (4 °C) CellCover reagent. Aliquots of 800 µL containing 4.5 × 10^6^ HEK/3T3 cells or 3.2 × 10^6^ MDMs were immediately stored at 4 °C until further processing according to the manufacturer´s instructions. After storage overnight at 4 °C some replicates were transferred to −20 °C storage in order to evaluate whether CellCover reagent also allows the preservation of frozen cells.

For scRNA-seq analysis samples stored at −20 °C were thawed and kept on ice throughout the procedure. Likewise, the samples stored at 4 °C were kept on ice during the procedure. Samples were centrifuged for 5 min at 300 g in a centrifuge pre-cooled to 4 °C and the CellCover supernatant was removed. Cell pellets were washed once in cold PBS/BSA buffer and then resuspended in the same buffer, filtered through a 40 µm cell strainer and transferred into the Drop-seq device.

### Library preparation and sequencing

Suitability of the protocols and reagents to preserve cells for scRNA-seq was evaluated using two popular droplet-based scRNA-seq systems, Drop-seq and the 10x Genomics Chromium device. Species mixing experiments and MDMs were processed by the Drop-seq system whereas the Chromium device was used for primary immune cells. In contrast to the Chromium device the Drop-seq platform did not support parallelised sample processing; therefore we prepared a single replicate per sample for the Drop-seq experiments. In order to allow comparability of the samples we minimised sources of technical bias by ensuring that cell handling and operation of the scRNA-seq devices were always performed by the same person. Additionally, all libraries were prepared by the same person and were pooled and sequenced on the same flowcell per experiment.

#### Drop-seq

The Drop-seq system (Dolomite Bio, Royston, UK) was operated as described by the manufacturer using cell suspensions of 200 cells/µL. Barcoded beads (Barcoded Bead SeqB, ChemGenes Corp., Wilmington, MA) were diluted to a concentration of 300 beads/µL. Droplets were collected in a 50 mL centrifuge tube (Sample Tube) and processed as described by Macosko *et al*.^6^ including minor modifications. During droplet breakage the first supernatant (after adding 30 mL 6X SSC and 1 mL perfluorooctanol to the collected droplets, shaking vigorously four times and centrifugation for 1 min at 1,000 g) was not discarded as described in the original protocol but collected in a 50 mL centrifuge tube (Supernatant Tube) and immediately centrifuged for 1 min at 1,000 g in order to increase the bead yield. Then, the supernatant was immediately discarded. Beads remaining on the oil interface in the Sample Tube were spun up using 30 mL of 6X SSC and transferred to the Supernatant Tube. Following another round of centrifugation for 1 min at 1,000 g the supernatant was removed and discarded. Beads were transferred to a 2 mL Eppendorf cup and washed twice with 1 mL of 6X SSC and then with 300 µL of 5x Maxima H-RT buffer (Thermo Fisher Scientific, Waltham, MA). After reverse transcription and exonuclease reaction the cDNA attached to beads was amplified during 4 + 10 and 4 + 11 PCR cycles for the species mixing experiment and MDMs, respectively. In total, 39,000 beads per sample were used for cDNA amplification and split into 13 PCR reactions using 3,000 beads each. All PCR reactions per sample were pooled and purified twice with 0.6 volumes of Agencourt AMPure XP beads (Beckman Coulter, Brea, CA). Quality of the cDNA was assessed by Fragment Analyzer analysis using 1 µL per sample and the High Sensitivity NGS Fragment 1–6000 bp Assay (AATI, Agilent Technologies, Santa Clara, CA).

Libraries were prepared from 670 pg of amplified cDNA using the Nextera XT DNA sample preparation kit (Illumina, San Diego, CA). During the final PCR reaction (12 cycles) custom primers and indices were used and the libraries were purified as described before^6^. Libraries were on average 611 bp and 646 bp in length for the species mixing experiment and MDMs, respectively. Paired-end sequencing was performed on a NextSeq 500 (Illumina, San Diego, CA) using a 75 cycles NextSeq™ 500 High Output Kit v2 (Illumina, San Diego, CA) and comprised a 20 bp read 1 (12 bp cell barcode and 8 bp UMI), 8 bp index read as well as a 50 bp read 2 (transcript sequence read). For the conversion of BCL files to FASTQs, we used bcl2fastq v2.17.1.14 with the parameters “–minimum-trimmed-read-length 20” and “–mask-short-adapter-read 1”.

#### 10x Genomics platform

Operation of the Chromium Controller and library preparation using the Single Cell 3’ Reagent Kits v2 (10x Genomics, Pleasanton, CA) were performed according to the instructions of the manufacturer. The Single Cell 3’ Chip was loaded aiming for 5,000 captured cells. cDNA from captured immune cells was amplified during cDNA amplification by 13 PCR cycles and the libraries were prepared from 110 ng of cDNA including 13 cycles of amplification during the final index PCR reaction. In addition to the cleanup procedure described by the manufacturer an additional cleanup step with 1 volume of SPRISelect Beads (Beckman Coulter, Brea, CA) was performed to remove excessive primers and primer dimers.

Libraries were on average 450 bp in length and sequencing was performed on a HiSeq 4000 (Illumina, San Diego, CA) using a HiSeq 3000/4000 PE Cluster Kit and two 50-cycle SBS kits (Illumina, San Diego, CA). Paired-end sequencing included a 26 bp read 1 (16 bp cell barcode and 10 bp UMI), 8 bp index read and 98 bp read 2 (transcript sequence read).

### Data processing

Sequencing reads from Drop-seq experiments were aligned using STAR aligner version 2.3.0^20^ and aligned against a merged reference of both human hg19 and murine mm10 for the species mixing experiment whereas reads of MDMs were aligned to GRCh38 and annotated according to Ensembl release 86^21^. The STAR genome indices were generated using the parameters “–sjdbOverhang 49” and “–genomeSAsparseD 2”. Drop-seq tools software^6^ version 1.12 was then applied to generate a digital expression matrix for each sample.

10x Chromium data were processed with Cell Ranger version 2.1.1^7^ and the Rnor6.0 reference genome was used to align the reads. Annotation of the reads was based on Ensembl release 86.

### Data analysis

In order to assess and compare the performance of the three preservation methods conserved cells were compared to fresh cells. The dropbead R package^8^ version 0.25 was applied to identify cell barcodes that represented real cells. Cells kept for further analysis were identified from a cumulative plot of read counts detected per cell barcode where the inflection point acts as a cutoff to distinguish background from proper single cells. Additionally, cell barcodes with less than 100 detected genes were excluded from further analyses to avoid a bias by background noise.

#### Cell impurity

Filtered species mixing single-cell data were used to estimate cell impurity for each cell barcode per sample. Cell impurity represents the fraction of transcripts per human cell that originate from murine cells and vice versa and covers cross-species contamination by ambient RNA as well as cross-species cell doublets. We performed Barnyard plot analysis as described elsewhere^6^ to identify the species of origin per cell. Cells with ≥50% of human transcripts were assigned as human cells while the remaining cells represented murine cells.

#### Quality control, single-cell clustering and cell annotation

The quality of fresh and preserved single cell samples was assessed by commonly used quality control parameters including the gene and UMI count as well as the percentage of mitochondrial transcripts per cell. These quality control parameters were calculated for cells selected by the dropbead package as part of the scRNA-seq data analysis procedure using the Seurat R package version 2.3.4^22^. The sample data sets corresponding to each of the species mixing, MDM and rat liver immune cell experiments were respectively merged into one R Data object per experiment for joint cluster analysis. In a next step the count data per cell were normalized and log transformed using the default settings of Seurat’s “NormalizeData” function. Principal component analysis and canonical correlation analysis were performed using the highly variable genes for the species mixing experiment and rat liver immune cells, respectively. The principal components and canonical vectors explaining the most variance (11 for the species mixing experiment and 20 for rat immune cells) were selected for subsequent cluster analysis and the immune cell data sets were aligned by animal of origin. Cluster analysis was computed at 0.2 and 0.4 resolution for the species mixing experiment and rat immune cells, respectively. Single-cell clustering was visualized by t-distributed stochastic neighbor embedding (t-SNE) plots using default parameters. Cell types of the rat immune cell samples were manually annotated by marker genes that were at least 2-fold increased in individual cell clusters compared to the remaining cells.

#### Similarity of fresh and preserved gene expression profiles

Statistically significant differences between gene levels of the fresh and preserved cells as well as between cell clusters was calculated by the MAST linear model approach, R package version 1.4.1^23^, as implemented in the Seurat package. Genes at least 2-fold deregulated were considered to be significantly altered if *P* values adjusted for multiple testing were less than 0.05 (Bonferroni correction).

Overall similarity of fresh and preserved “pseudo-bulk” gene expression profiles was assessed by correlation and hierarchical cluster analysis. Pseudo-bulk profiles were generated by calculating the sum of the transcript counts across all cells per sample. The raw pseudo-bulk count matrices were scaled and the expression levels recalculated into counts per million using edgeR^24^ version 3.20.9. Pearson correlation of the fresh and preserved samples was computed by means of the dropbead package using the filtered count matrices as input which contained cell barcodes that represented real cells. For hierarchical cluster analysis, the pheatmap package version 1.0.8^25^ was applied to the log_2_ transformed pseudo-bulk profiles using default parameters and the entire gene set per sample. A pseudo-count of 0.5 was added per gene count prior to log_2_ transformation.

To identify genes that were affected by storage duration we performed time course analysis for gene expression over time using the limma R package^26^. Raw single-cell as well as pseudo-bulk gene count matrices were processed into counts per million (CPM) and analysed using linear models that were fitted using the lmFit function of limma with time added as a factor in the design matrix for 0 (for fresh), 1 week, and 15 weeks. Statistics were calculated by empirical Bayes moderation and genes were considered to be affected by preservation if FDR adjusted *P* values were <0.05 and a fold change ≥2 in either direction.

For the species mixing experiment all analyses were performed separately for both, human and murine cells to capture differences between the two cell lines.

## Results

### Systematic comparison of cell preservation protocols

#### Cell integrity and cell impurity are highly variable across protocols

**Figure 1:**
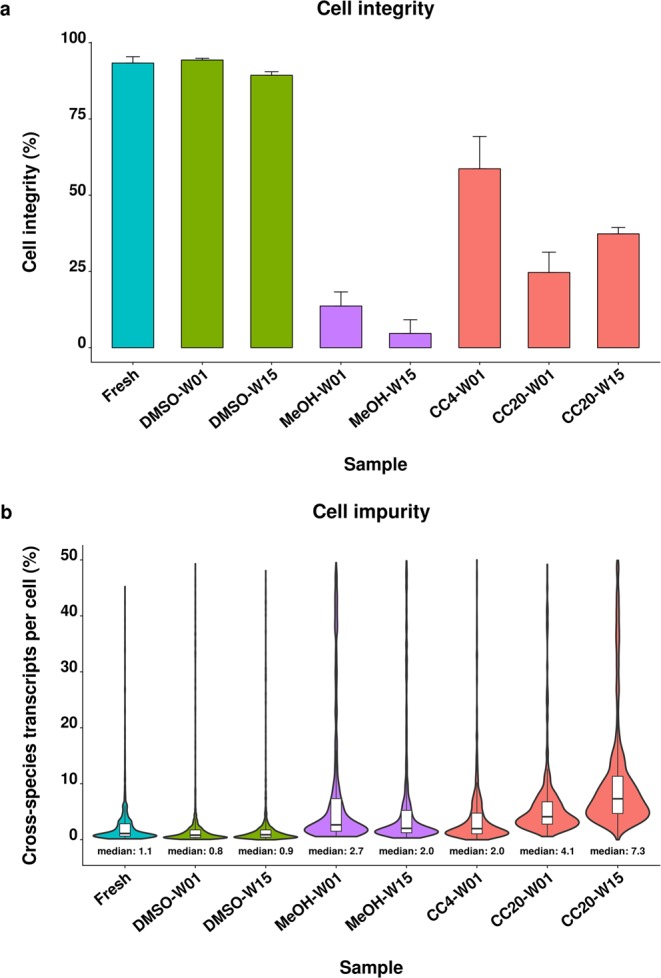
Cell integrity and cell impurity of fresh and preserved cells. Cell integrity (**a**) and cell impurity (**b**) were determined for the fresh human/mouse cell mixture and cells preserved using DMSO cryopreservation (DMSO), methanol fixation (MeOH) and CellCover reagent at 4 °C (CC4) and −20 °C (CC20). Cells were stored for one (W01) and 15 weeks (W15). Cell integrity is represented by the percentage of undamaged cells as determined by live/dead staining. Cell impurity reflects the fraction of cross-species transcripts per cell barcode including contamination by ambient RNA as well as co-encapsulated cells.

In order to compare protocols for scRNA-seq compatible cell preservation we performed a species mixing experiment using a mixture of human and murine cells on the Drop-seq platform. First, the integrity and cell impurity of the preserved cells were investigated to compare the different protocols. The fresh cells contained mainly living cells indicated by a cell integrity measure of 93%. DMSO cryopreservation maintained high cell integrity of 94% and 89% for the cells stored for one and 15 weeks, respectively. In contrast, cell integrity dropped substantially below 15% after methanol fixation for one and 15 weeks. Similarly, cell integrity of the samples preserved by CellCover reagent declined to 59%, 25% and 37% after storage at 4 °C for one week and at −20 °C for one and 15 weeks, respectively (Fig. 1a).

Cell impurity, defined as the fraction of transcripts per human cell that originated from murine cells and vice versa, was similar for fresh and DMSO preserved cells indicated by a median cell impurity of 0.8–1.1%. Methanol preservation resulted in a higher fraction of cells with increased cell impurity exemplified by an approximately 2-fold increased median cell impurity of 2.0–2.7%. Cell impurity was most variable for the cells stored in CellCover reagent indicated by medians of 2.0% up to 7.3% for the cells stored for one week at 4 °C and 15 weeks at −20 °C, respectively (Fig. 1b).

### Impact of cell preservation on scRNA-seq quality control parameters

In addition to cell integrity and cell impurity, scRNA-seq quality control parameters were investigated on 603–1,553 cells per sample (Supplementary Table S1) to further assess the performance of the preservation protocols. First, we investigated the percentage of mitochondrial transcripts as well as the number of genes and UMIs detected per cell which are commonly used parameters to assess the quality of scRNA-seq samples.

**Figure 2:**
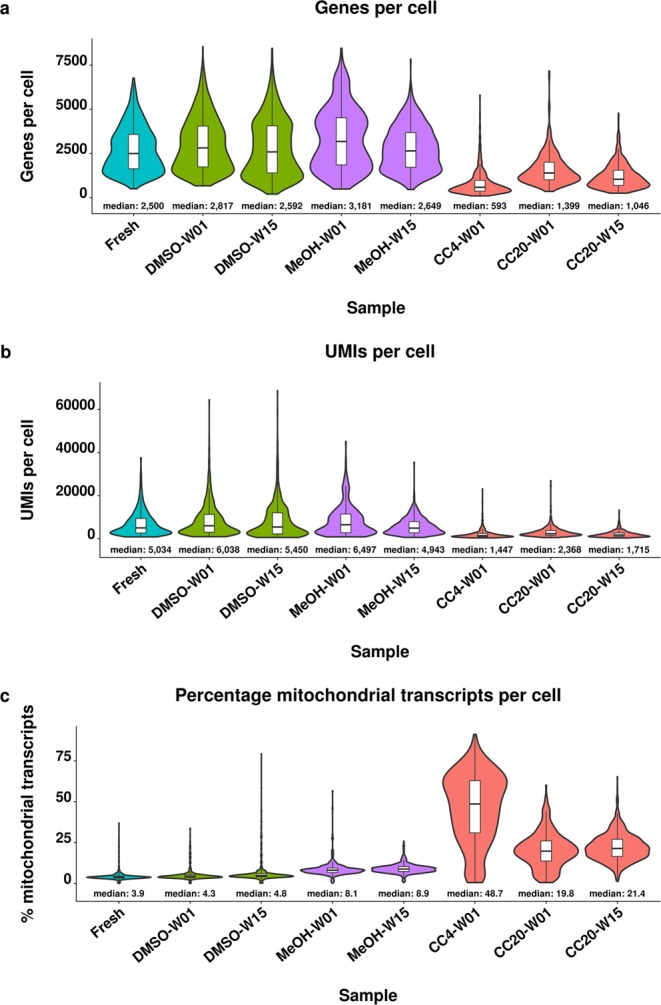
Quality control parameters of fresh and preserved human cells. Distribution and median gene count per cell (**a**), unique molecular identifier (UMI) count per cell (**b**) and percentage of mitochondrial transcripts per cell (**c**) detected for fresh cells and after DMSO cryopreservation (DMSO), methanol fixation (MeOH) and storage in CellCover reagent at 4 °C (CC4) and −20 °C (CC20). Cells were stored for one (W01) and 15 weeks (W15). Data shown are derived from human HEK293 cells of the species mixing experiment.

The median numbers of gene counts per cell were similar for fresh cells and cells preserved by DMSO cryopreservation as well as methanol fixation, ranging between 2,500 and 3,181 genes for human cells (Fig. 2a). Likewise, the median number of UMI counts detected per human cell was in the similar range and between 4,943 and 6,497 UMIs per cell (Fig. 2b). Both, the number of genes and UMIs detected per cell were decreased in cells that were preserved using the CellCover reagent. Median gene and UMI counts per cell were 1.8-fold to 4.2-fold and 2.1-fold to 3.5-fold decreased compared to fresh cells, respectively (Fig. 2a,b). In contrast, the median percentage of mitochondrial transcripts was 5.1-fold to 12.5-fold increased after CellCover preservation and approximately 2-fold increased for methanol fixed cells. The median percentage of mitochondrial transcripts of 3.9% to 4.8% was similar for DMSO cryopreserved and fresh cells (Fig. 2c). The patterns observed for human cells were similar in the murine cells of the species mixing experiment (Supplementary Fig. S1).

### DMSO cryopreservation maintains highly similar gene expression profiles

**Figure 3:**
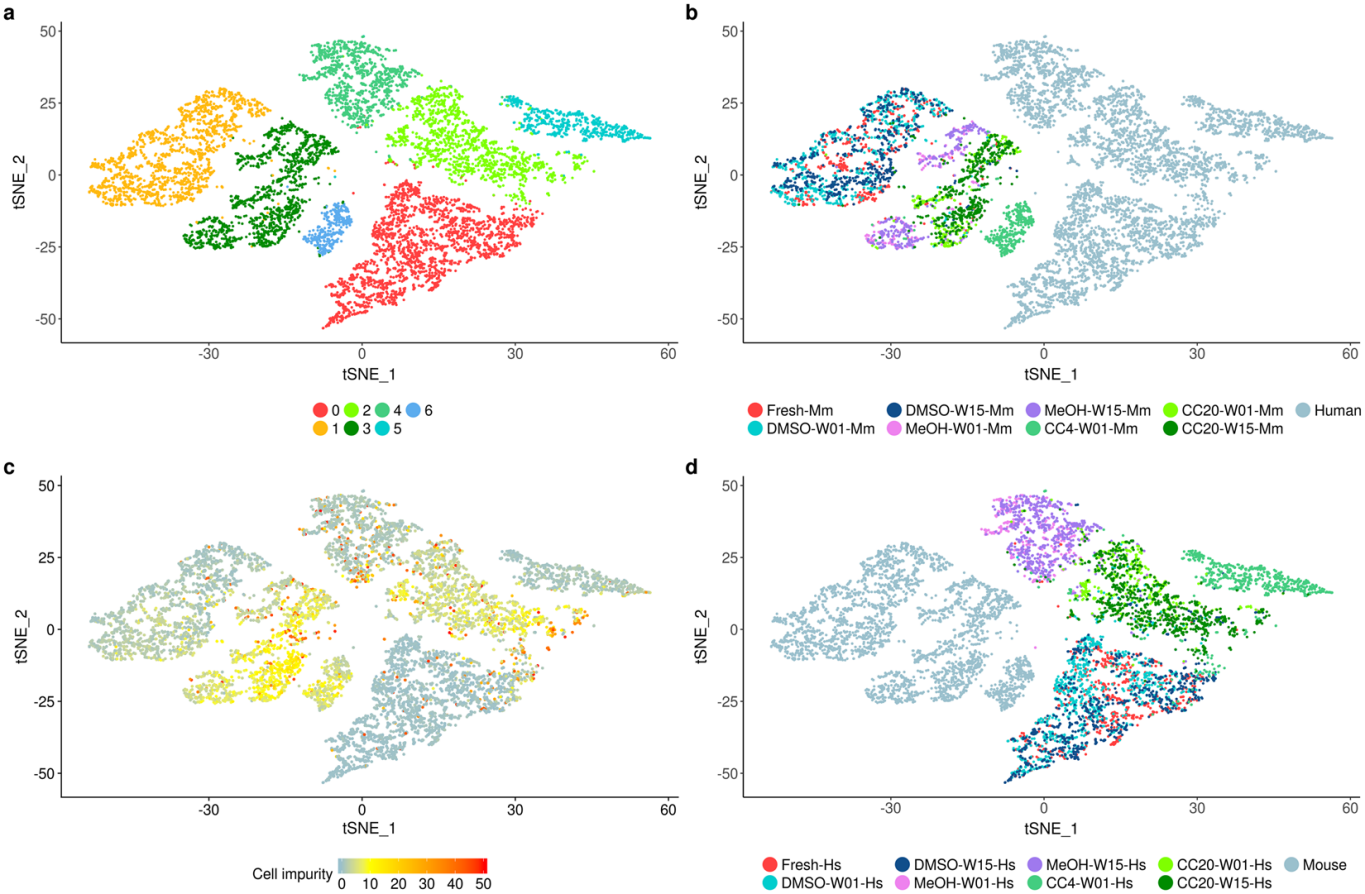
Cluster analysis of fresh and preserved species mixing samples. Cell clustering identified seven cell clusters (**a**) and was mainly driven by murine (Mm) and human (Hs) origin and the preservation method. DMSO cryopreserved (DMSO) cells clustered together with the fresh cells whereas the cells preserved by CellCover reagent at 4 °C (CC4) and −20 °C (CC20) as well as methanol fixation (MeOH) formed separate clusters for murine and human cells respectively (**b**,**d**). The fraction of cross-species transcripts per cell (“cell impurity”) was variable across the different protocols and species (**c**). W01, storage for one week; W15, storage for 15 weeks.

We further investigated the impact of preservation on cellular and pseudo-bulk expression profiles. Cluster analysis revealed seven cell clusters that were driven by human (HEK293 cells) or murine (3T3 cells) cell origin and the preservation method (Fig. 3). The fresh and DMSO preserved cells clustered together into one distinct cell cluster per species indicating that the expression profiles of the fresh cells were most similar to DMSO preserved cells (Fig. 3b,d). In contrast, cells preserved by CellCover reagent at 4 °C formed separate clusters for both species. Similarly, human cells stored in CellCover reagent at −20 °C and methanol fixed cells formed separate clusters whereas the respective murine cells clustered within one cell cluster (Fig. 3a,b,d). Most human and murine cells preserved by CellCover reagent at −20 °C showed a moderate cross-species contamination of 5–10% (Figs 1b and 3c). For the cells preserved by the remaining protocols the fraction of cross-species contaminated cells was considerably lower but murine cells seemed to be more affected by cross-species contamination than human cells. This pattern was particularly observed for MeOH fixed cells and cells preserved by CellCover reagent at 4 °C whereas the fraction of cross-species contaminated fresh and DMSO preserved cells was lower (Fig. 3b–d).

Single-cell cluster analysis identified differences between fresh and preserved cells based on the highly variable genes. The similarity of the preserved cells relative to the fresh cells was also investigated on the entire gene set by hierarchical cluster analysis of the pseudo-bulk (aggregated) profiles from the same samples. The pseudo-bulk hierarchical clustering reiterated the clustering patterns observed on the single-cell level. Pseudo-bulk gene expression profiles of DMSO preserved cells showed the highest similarity to fresh cells whereas methanol fixed cells and cells preserved by CellCover reagent clustered separately (Supplementary Fig. S2).

**Figure 4:**
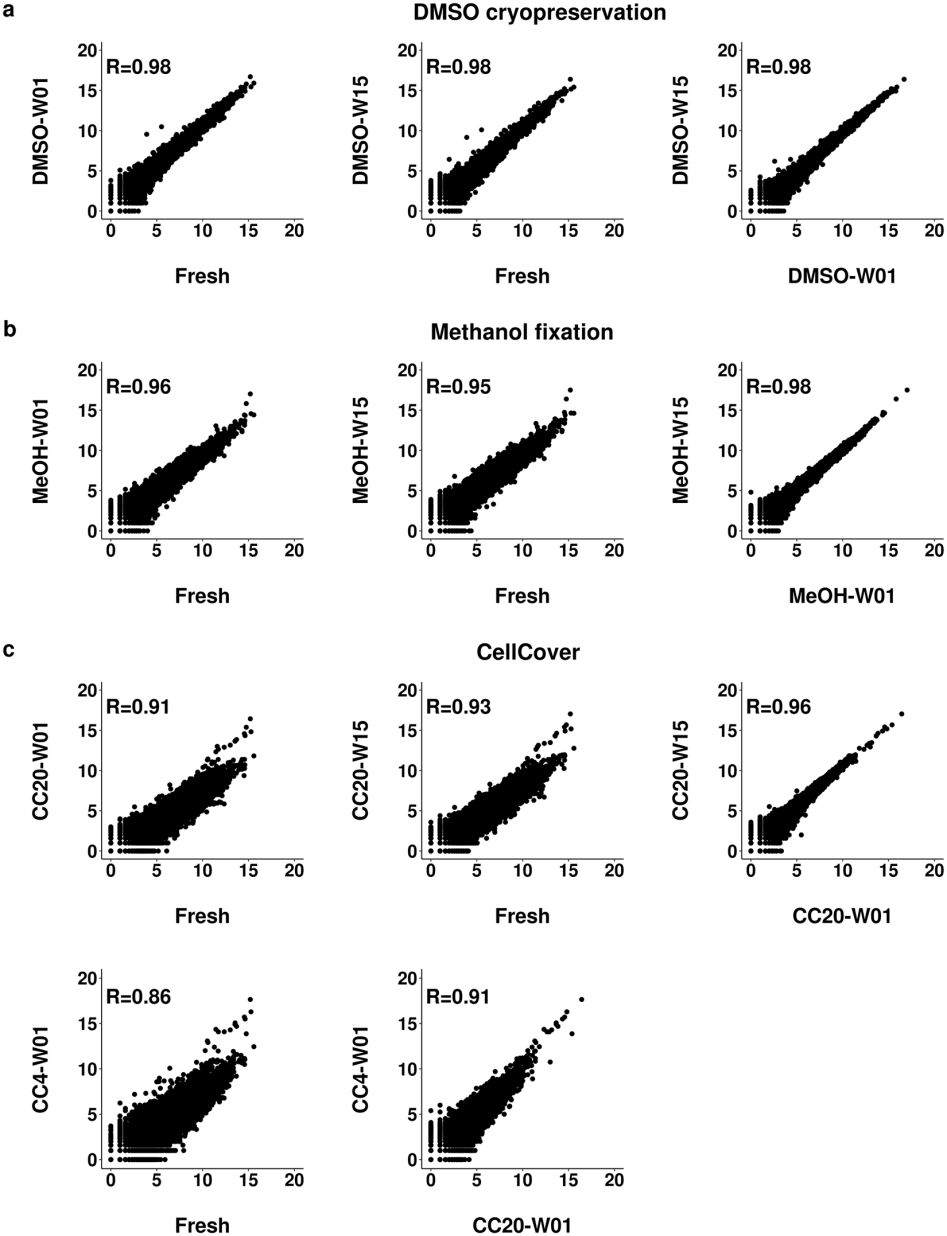
Pseudo-bulk gene expression correlation of fresh and preserved cells. Scatter plots show pairwise correlation of pseudo-bulk gene expression profiles from fresh cells and DMSO cryopreserved (DMSO) cells (**a**), methanol fixed (MeOH) cells (**b**) and cells preserved by CellCover reagent (**c**) at 4 °C (CC4) and −20 °C (CC20). Cells were stored for one week (W01) and 15 weeks (W15). Pearson correlation coefficient (R) indicates the degree of correlation. Data are shown for human HEK293 cells derived from the species mixing experiment. Axes represent log_2_ (UMI + 1) counts.

Additionally, pseudo-bulk expression profiles of preserved and fresh cells were compared using correlation scatter plots. DMSO preserved cell profiles correlated best with those of fresh cells (R = 0.98 and R = 0.97 in human and mouse samples respectively), independently of storage duration. Methanol fixation (R = 0.95–0.96) was slightly worse and samples stored in CellCover (R = 0.90–0.93 at −20 °C and R = 0.86 at 4 °C) were least-well preserved (Fig. 4, Supplementary Fig. S3).

We also examined whether storage duration affects gene expression. Pseudo-bulk gene expression profiles of the cells preserved for one and 15 weeks were highly correlated (R ≥ 0.96 for human and murine cells respectively) independent of the protocol (Fig. 4, Supplementary Fig. S3). A time series analysis using the linear model “lmFit” function from the limma R package revealed that the gene expression profiles in either the single cell or pseudo-bulk scenarios were not significantly altered along the storage time period when assessed with an adj. *P* < 0.05 and a fold change ≥2.

Analysis of the statistically significant differences between the gene levels of fresh and preserved cells confirmed high similarity of the fresh and DMSO preserved cells with only 8 and 7 significantly altered (FC ≥2, adj. *P* < 0.05) genes in human HEK293 cells stored for one and 15 weeks, respectively. Similarly, altered levels of 15 and 25 genes were identified for murine 3T3 cells after one and 15 weeks of storage. Comparison of the significantly altered genes across species and both storage periods revealed a consistent increase of *FOS* and *FOSB* levels in DMSO preserved cells. The number of significantly altered genes was increased for methanol fixation (25–82 genes per sample). Consistently increased levels of the mitochondrial transcripts *MT*-*RNR1* and *MT*-*RNR2* were found for methanol fixed cells independent of the species and storage period. The CellCover preserved cells were most distinct from fresh cells illustrated by 135 to 813 significantly altered genes (Supplementary Table S2). Increased levels of several mitochondrial transcripts, an indicator of dying cells and decreased cell quality, were observed for CellCover preserved cells and up to 21.8-fold (*MT*-*RNR1*) increased for the sample stored at 4 °C.

### Protocol performance is highly variable for monocyte-derived macrophages

**Figure 5:**
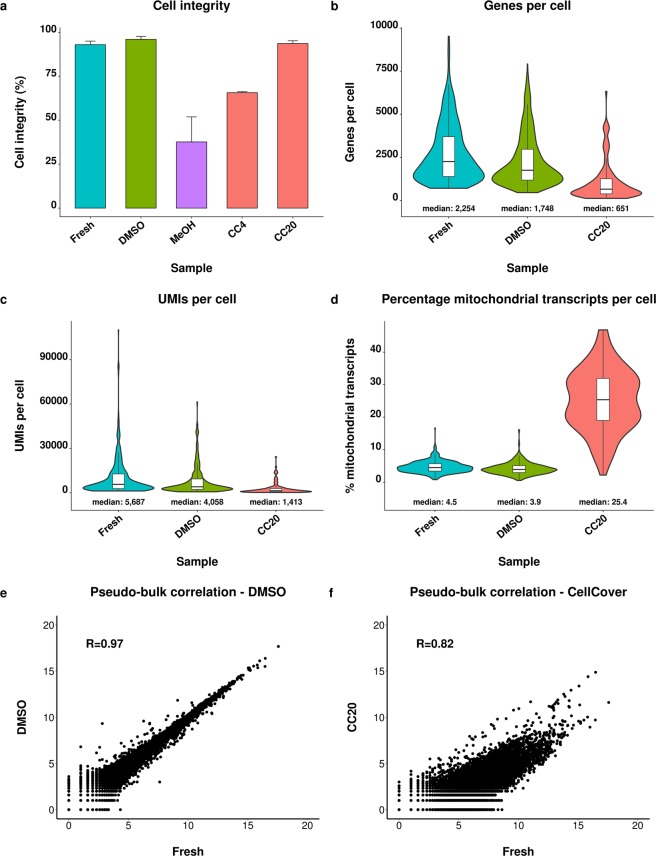
Comparison of fresh and preserved monocyte-derived macrophages. Comparison of cell integrity (**a**), gene count per cell (**b**), unique molecular identifier (UMI) count per cell (**c**), percentage of mitochondrial transcripts per cell (**d**) and pearson correlation of pseudo-bulk gene expression profiles (log_2_ (UMI + 1) counts) from fresh and preserved cells (**e**,**f**). Cells were preserved by DMSO cryopreservation (DMSO), methanol fixation (MeOH) and CellCover reagent at −20 °C (CC20). The capture of cDNA from methanol preserved MDMs and processing of MDMs stored in CellCover at 4 °C was not feasible, therefore the analysis of these preservation methods is not presented. Error bars indicate the standard deviation.

In addition to cell lines, the protocols were tested for primary cell derived monocyte-derived macrophages using the Drop-seq platform (127–571 cells per sample, Supplementary Table S1). The integrity of DMSO cryopreserved cells and cells stored in CellCover reagent at −20 °C was similar to fresh cells being between 93% and 96%. Cell integrity decreased substantially to 66% and 36% upon storage in CellCover reagent at 4 °C and by methanol fixation, respectively (Fig. 5a).

Interestingly, we did not succeed to obtain cDNA from methanol preserved MDMs although the presence of cellular entities in the preserved sample was confirmed during cell counting. Processing of a second replicate of the methanol fixed MDMs confirmed the finding and also did not yield any cDNA (data not shown). Additionally, scRNA-seq analysis could not be performed for MDMs recovered from CellCover storage at 4 °C since most cells were lost during storage and the final cell count was insufficient to perform scRNA-seq analysis.

The expression profiles of DMSO cryopreserved MDMs were similar to fresh MDMs indicated by similar but slightly decreased gene and UMI counts per cell as well as strongly correlated (R = 0.97) pseudo-bulk expression profiles (Fig. 5b,c,e). Cells preserved by the CellCover reagent were characterized by strongly decreased UMI and gene counts as well as a 5.6-fold increased abundance of mitochondrial transcripts (Fig. 5b–d). As expected, pseudo-bulk profiles of MDMs stored in the CellCover reagent were less similar (R = 0.82) to fresh cells compared to MDMs preserved by DMSO cryopreservation (R = 0.97, Fig. 5e,f).

The high similarity between the pseudo-bulk profiles of DMSO cryopreserved and fresh MDMs was confirmed by as little as 6 significantly (adj. *P* < 0.05, fold change ≥2) altered genes (Supplementary Table S3). In contrast, the levels of 506 genes were significantly different compared to fresh cells after preservation by the CellCover reagent which is in line with the decreased overall correlation of the pseudo-bulk profiles (Fig. 5e,f). Interestingly, *FOS* and *FOSB* were 3.8-fold and 2.8-fold increased by DMSO cryopreservation respectively, which has already been observed in the species mixing experiment. Mitochondrial transcripts were strongly increased (up to 23-fold) in CellCover preserved cells which is also consistent with the species mixing experiment.

### DMSO cryopreservation allows the preservation of primary immune cells

#### Fresh and cryopreserved gene expression profiles are highly similar

Finally, we investigated whether our initial findings are validated in a different cell population and on a different platform for the preservation method that performed best. We applied the DMSO cryopreservation protocol to immune cells enriched from rat liver and performed the experiment on the Chromium system which enabled the analysis of 3,542–4,719 cells per sample (Supplementary Table S1).

**Figure 6:**
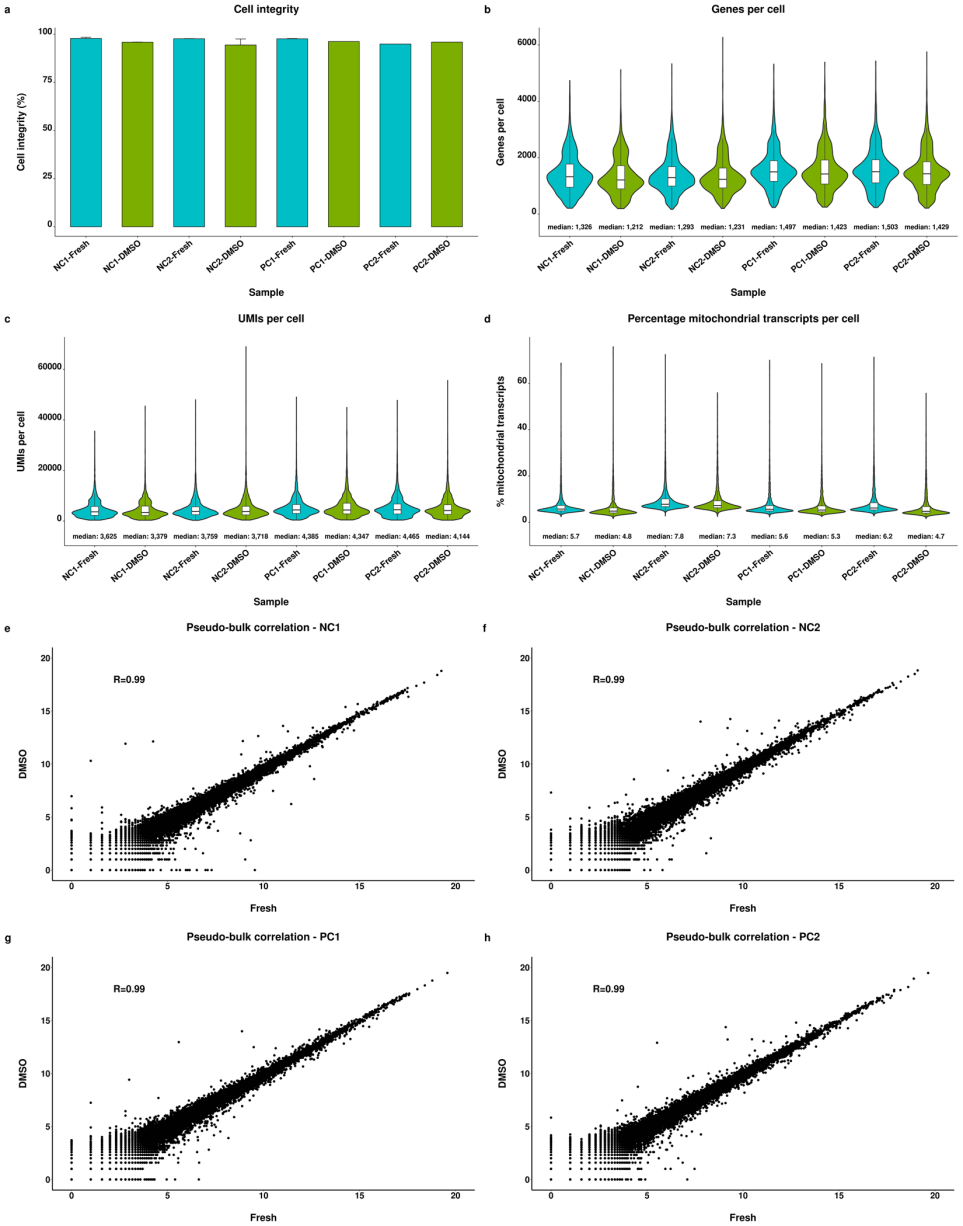
Comparison of fresh and preserved immune cells. Comparison of cell integrity (**a**), gene count per cell (**b**), unique molecular identifier (UMI) count per cell (**c**) and percentage of mitochondrial transcripts per cell (**d**) of fresh immune cells isolated from rat liver and after storage using the DMSO cryopreservation protocol. Scatter plots show correlation of pseudo-bulk gene expression profiles with axes representing log_2_ (UMI + 1) counts (**e**–**h**). Cells were isolated from two negative (NC) and positive control (PC) animals, respectively. Error bars indicate the standard deviation.

The DMSO cryopreservation protocol maintained a highly viable cell population indicated by approximately 95% of living cells, which was similar to fresh cells (Fig. 6a). The median gene counts per cell were highly similar for fresh and preserved samples and were in a range between 1,200 to 1,300 genes for NC animals and 1,400 to 1,500 genes for PC animals (Fig. 6b). The same pattern was also observed for UMI counts indicated by median values of 3,379 to 3,759 and 4,144 to 4,465 for NC and PC derived samples, respectively (Fig. 6c). The percentage of mitochondrial transcripts was also highly similar for fresh and preserved samples being between 5% and 8% (Fig. 6d). Additionally, barcode rank plots of the samples did not indicate an increased ambient RNA contamination of the DMSO preserved cells (Supplementary Fig. S4).

Comparison of the gene expression patterns showed that fresh and preserved samples were highly similar indicated by strongly correlated pseudo-bulk profiles per animal of R = 0.99 (Fig. 6e–h). This finding was further substantiated by the low number of significantly altered gene levels between fresh and preserved cells of the same animal ranging between 7 and 14 genes (Supplementary Table S4). Consistent with cryopreserved MDMs and human as well as murine cell lines, significantly increased levels of both, *Fos* and *Fosb* and additionally *Jun* were observed for cryopreserved cells of all animals.

Hierarchical cluster analysis of the pseudo-bulk profiles revealed that the fresh and preserved cells originating from the same animals were more similar to each other than the fresh and DMSO preserved samples across animals (Supplementary Fig. S5). Therefore, inter-individual differences can be conserved by DMSO cryopreservation, demonstrating that this protocol barely affects the expression profiles of preserved cells.

### Immune cell populations are conserved by DMSO cryopreservation

**Figure 7:**
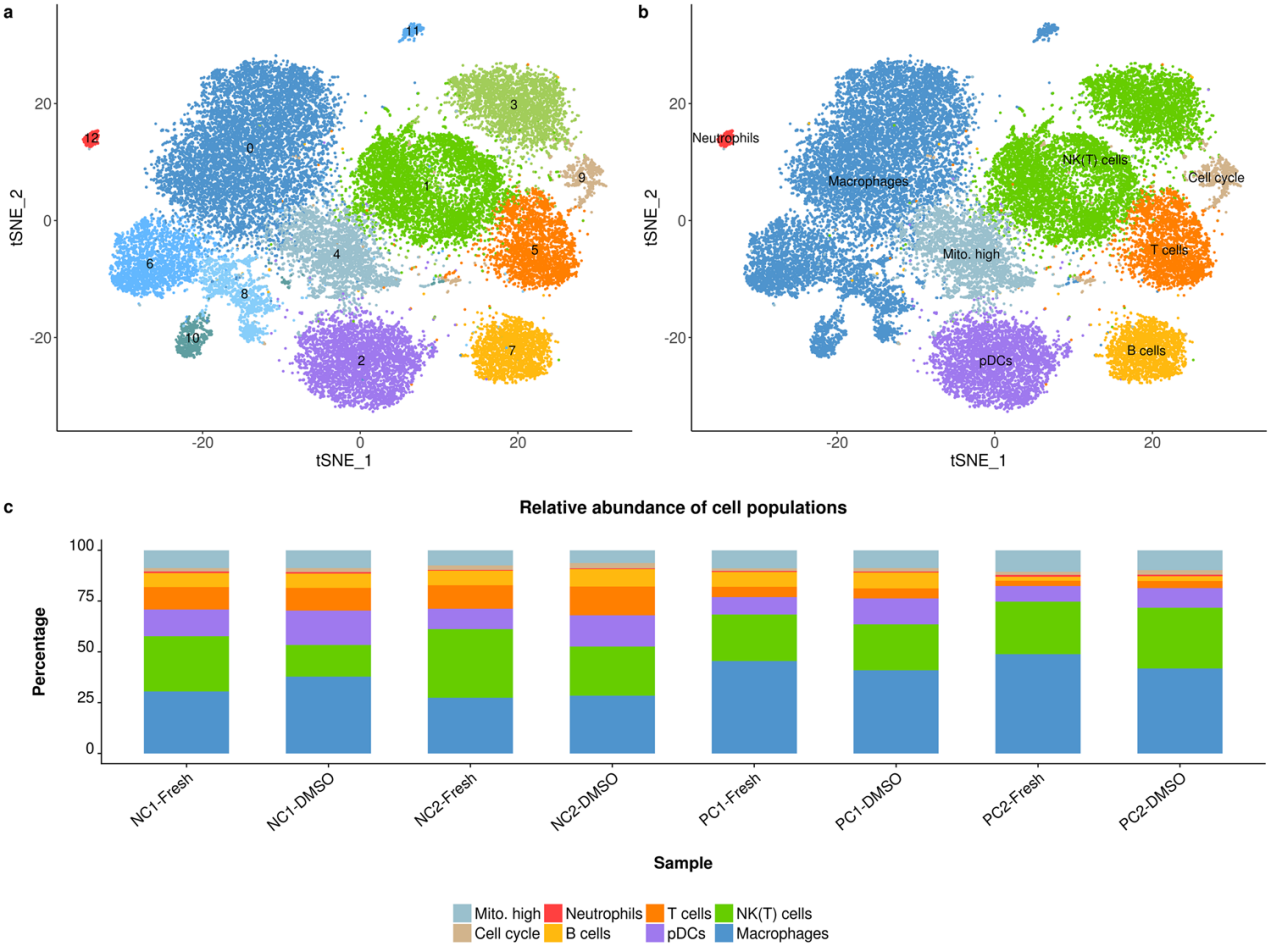
Immune cell populations in fresh and cryopreserved samples. Cluster analysis identified 13 cell clusters in immune cells isolated from rat liver (**a**). Cell clusters represent macrophages/Kupffer cells, natural killer/natural killer T cells (NK(T) cells), T cells, B cells, neutrophils, plasmacytoid dendritic cells (pDCs), cycling cells (Cell cycle) and cells with increased expression of mitochondrial transcripts (Mito. high, **b**). The relative abundance per cell population is shown for fresh and DMSO cryopreserved (DMSO) samples per negative control (NC) and positive control (PC) animal, respectively (**c**).

Cluster analysis on the single-cell level identified several cell types including five macrophage/Kupffer cell populations, two cell populations that expressed natural killer and natural killer T cell markers, T cells, B cells, neutrophils and plasmacytoid dendritic cells. Two more cell clusters were characterized by their cell cycle state and increased expression of mitochondrial transcripts (Fig. 7a,b).

All cell populations detected in fresh samples were also present after DMSO cryopreservation. Comparison of the relative abundance per cell type from fresh and cryopreserved samples revealed that DMSO cryopreservation maintained a highly similar cell population although there was some variability across the different animals (Fig. 7c). Interestingly, the cell population frequency in fresh and preserved samples was more similar for the positive than for the negative control animals but still strongly correlated for all animals (0.89≤ R ≤0.99; Supplementary Fig. S6).

## Discussion

Advances in scRNA-seq protocols have led to a rapid increase in scRNA-seq studies that contribute to the better understanding of several biological systems. Most studies still use fresh samples^27^ which is particularly true for droplet-based scRNA-seq systems. Recently, studies have shown that cell preservation protocols are compatible with scRNA-seq^8–10,13^ but there has been no comparative study to assess their suitability for droplet-based scRNA-seq. Here, we compared the most popular DMSO cryopreservation and methanol fixation methods, and for the first time the CellCover reagent using a species mixing experiment and MDMs to include a more difficult to preserve cell type.

Droplet-based scRNA-seq systems are particularly prone to biases by co-encapsulated cells as well as cell-free (“ambient”) RNA of damaged or dead cells that may cause misleading biological interpretation^28^. These contaminations should therefore be reduced to a minimum and not increased by preservation. We demonstrated that cell integrity and the independently determined cell impurity (“contamination of cells by cross-species transcripts”) was comparable for DMSO cryopreserved and fresh cells. Increased contamination of cells, as observed for methanol fixed and CellCover preserved cells, might therefore result in noisier data as compared to fresh and DMSO preserved cells. We would like to point out that the cell impurity metric does not include contamination by transcripts of the same species, which is another source of single-cell contamination that could not be directly quantified in our study.

Superior overall performance of the DMSO protocol was confirmed by a comparison of gene expression profiles demonstrating that this protocol allows preservation of cells that are highly similar to fresh cells. Methanol fixation was the second-ranked protocol and characterized by an increased percentage of mitochondrial transcripts, a measure for low quality cells, which is consistent with previous reports^8^. Similar gene counts per cell of fresh, DMSO preserved and methanol fixed cells as observed in our and previous studies^8,9^ indicate that both methods do not affect the complexity of preserved single-cell libraries. A decrease in gene counts and an increased percentage of mitochondrial transcripts per cell points towards the CellCover reagent being the worst performer for cell preservation.

Interestingly, methanol fixation failed to yield any cDNA from preserved MDMs. The inability of methanol fixation to preserve distinct cell types or tissues has been demonstrated before^8^ and was expected to be challenging for tissues that are rich in RNases and proteases including immune tissues^8^. Macrophages and MDMs express RNases^29,30^ which may explain the failure of methanol fixation to preserve these cells. A note stating that not all cell types may be preserved by CellCover reagent in the manufacturer’s instructions might explain the worse performance of the reagent for MDMs as compared to the species mixing experiment.

Next, we evaluated DMSO cryopreservation for heterogeneous primary immune cells which are commonly investigated in scRNA-seq studies to provide a comprehensive assessment of the most robust protocol. Recently, Chen *et al*. reported that methanol fixation is problematic for neutrophils^13^. Our data demonstrate that DMSO cryopreservation allows conservation of various immune cells including neutrophils which indicates wider applicability as compared to methanol fixation. However, DMSO cryopreservation is more labour-intensive than methanol fixation and preservation by CellCover and clearly a drawback of this protocol. Any preservation method will cause some slight perturbations to the cells, and we observed increased levels of *FOS*, *FOSB* and *JUN* in DMSO preserved cells across all evaluated cell matrices, suggesting that this protocol introduces a minor systematic bias. The JUN and FOS protein families regulate different cellular processes such as proliferation, apoptosis and cellular stress^31,32^ which has to be considered when applying this protocol. The consistency of this systematic bias across independent experiments, species, and platforms indicates that this is not a random process in the preservation of cells with DMSO. As a consequence studies should not compare across fresh and preserved cells although the DMSO protocol allows preservation of highly similar gene expression profiles. Furthermore, this consistent signal indicates that we captured relevant signals from our Drop-seq data although we were not able to analyse replicates for the Drop-seq experiments which is a limitation of our study.

Collectively, DMSO cryopreservation presents a highly robust protocol that broadly facilitates the preservation of many cell types, minimises cell contamination by foreign transcripts and consistently preserves highly similar gene expression profiles relative to the fresh cells.

Our work provides a useful understanding of the advantages and disadvantages among the different preservation protocols that can help researchers when deciding about cell preservation methods in their own study designs.

## Supplementary information

Supplementary information

## Data Availability

The RNA-seq data generated during the current study are deposited in Gene Expression Omnibus and accessible through accession number GSE127249.
