# Supplementary material for: DMSO cryopreservation is the method of choice to preserve cells for droplet-based single-cell RNA sequencing
Source: Sci Rep. 2019 Jul 23;9:10699. doi: 10.1038/s41598-019-46932-z (PMC6650608; doi:10.1038/s41598-019-46932-z)
Supplement: Supplementary file 1 — Supplementary information [file 41598_2019_46932_MOESM1_ESM.docx]

**Supplementary Information**

**DMSO cryopreservation is the method of choice to preserve cells for droplet-based single-cell RNA sequencing**

Christian T. Wohnhaas, Germán G. Leparc, Francesc Fernandez-Albert, David Kind, Florian Gantner, Coralie Viollet, Tobias Hildebrandt, Patrick Baum

**Supplementary Figures**

**Supplementary Figure S1.**


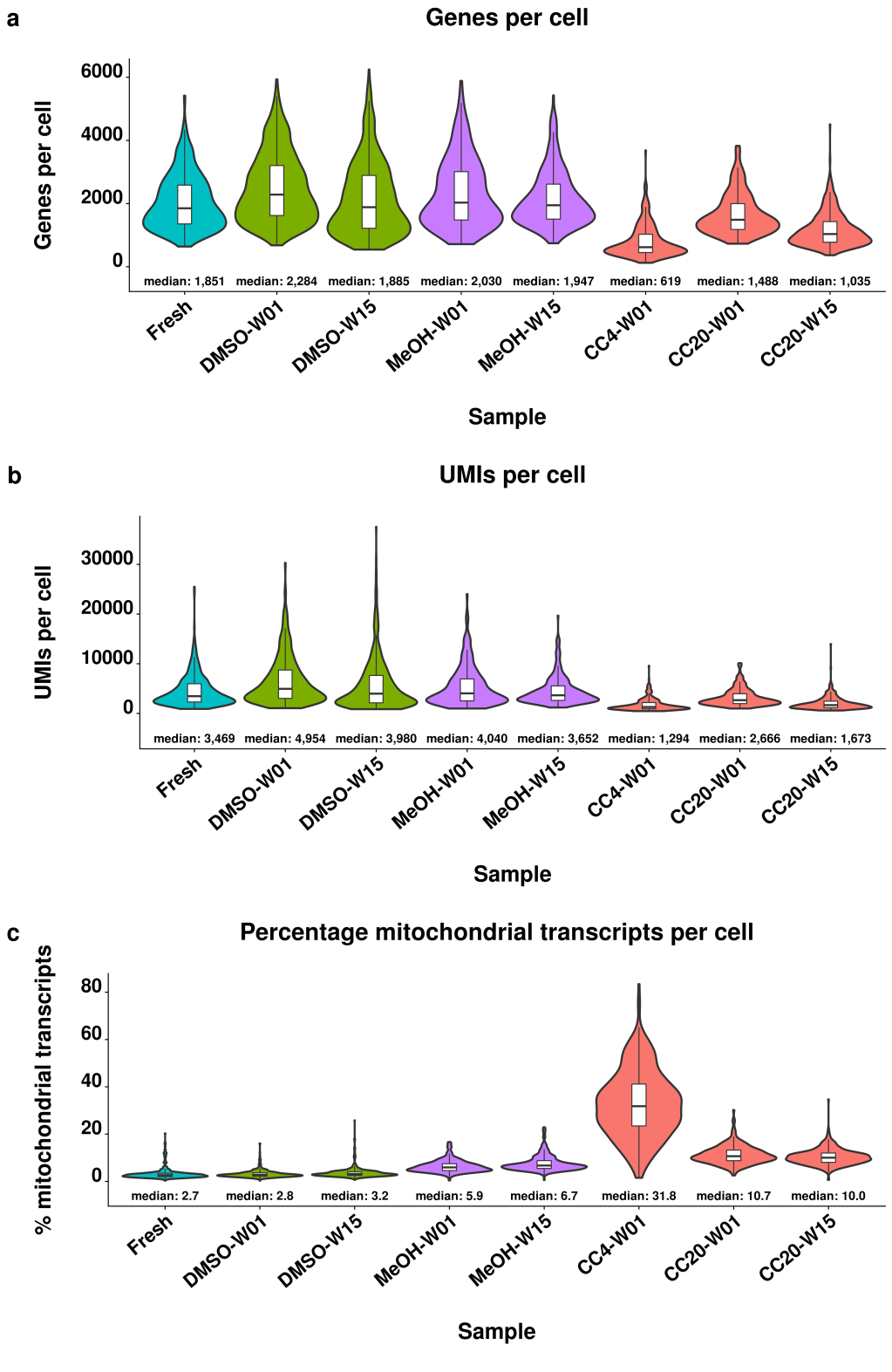


**Supplementary Figure S1. Quality control parameters of fresh and preserved murine 3T3 cells.**

Distribution and median gene count per cell (a), unique molecular identifier (UMI) count per cell (b) and percentage of mitochondrial transcripts per cell (c) detected for fresh cells and after DMSO cryopreservation (DMSO), methanol fixation (MeOH) and storage in CellCover reagent at 4 °C (CC4) and -20 °C (CC20). Cells were stored for one (W01) and 15 weeks (W15). Data shown are derived from murine 3T3 cells of the species mixing experiment.

**Supplementary Figure S2.**


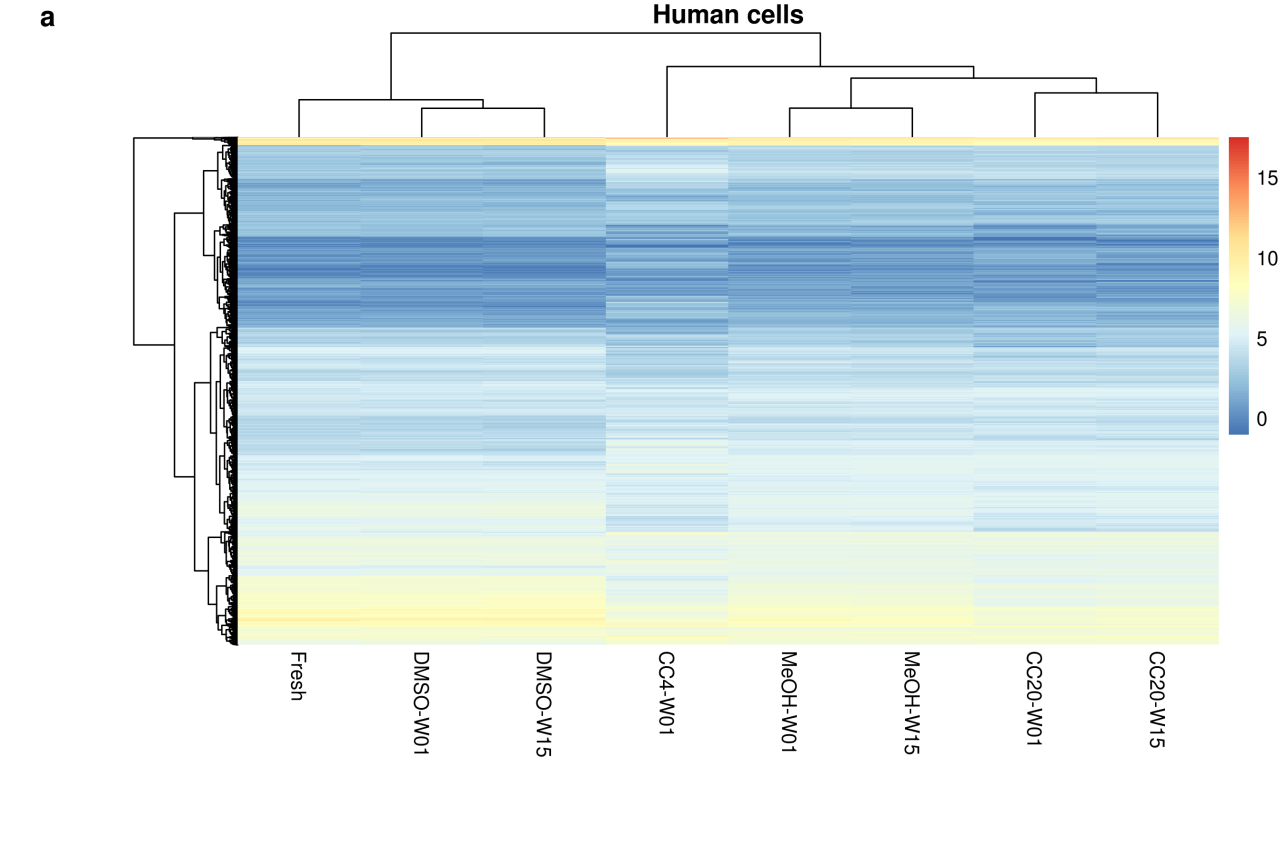


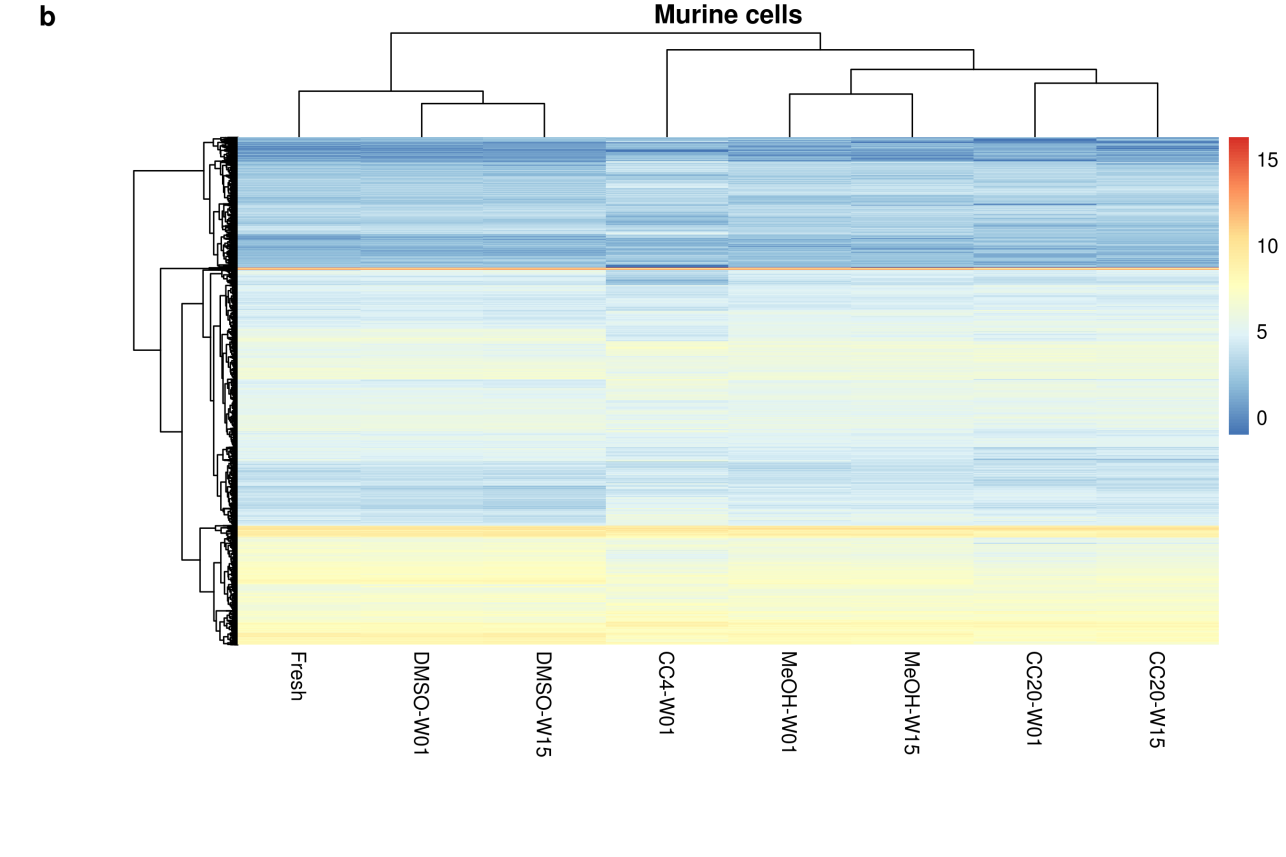


**Supplementary Figure S2. Hierarchical clustering of pseudo-bulk gene expression profiles.**

Hierarchical cluster analysis of pseudo-bulk gene expression profiles from the species mixing experiment. Hierarchical clustering based on the entire gene set is shown for fresh and preserved human HEK293 cells (a) and murine 3T3 cells (b). Cells were preserved by DMSO cryopreservation (DMSO), methanol fixation (MeOH) and CellCover reagent at 4 °C (CC4) and -20 °C (CC20) for one week (W01) and 15 weeks (W15), respectively. Color-coded gene expression levels are displayed in log_2_(counts per million).

**Supplementary Figure S3.**


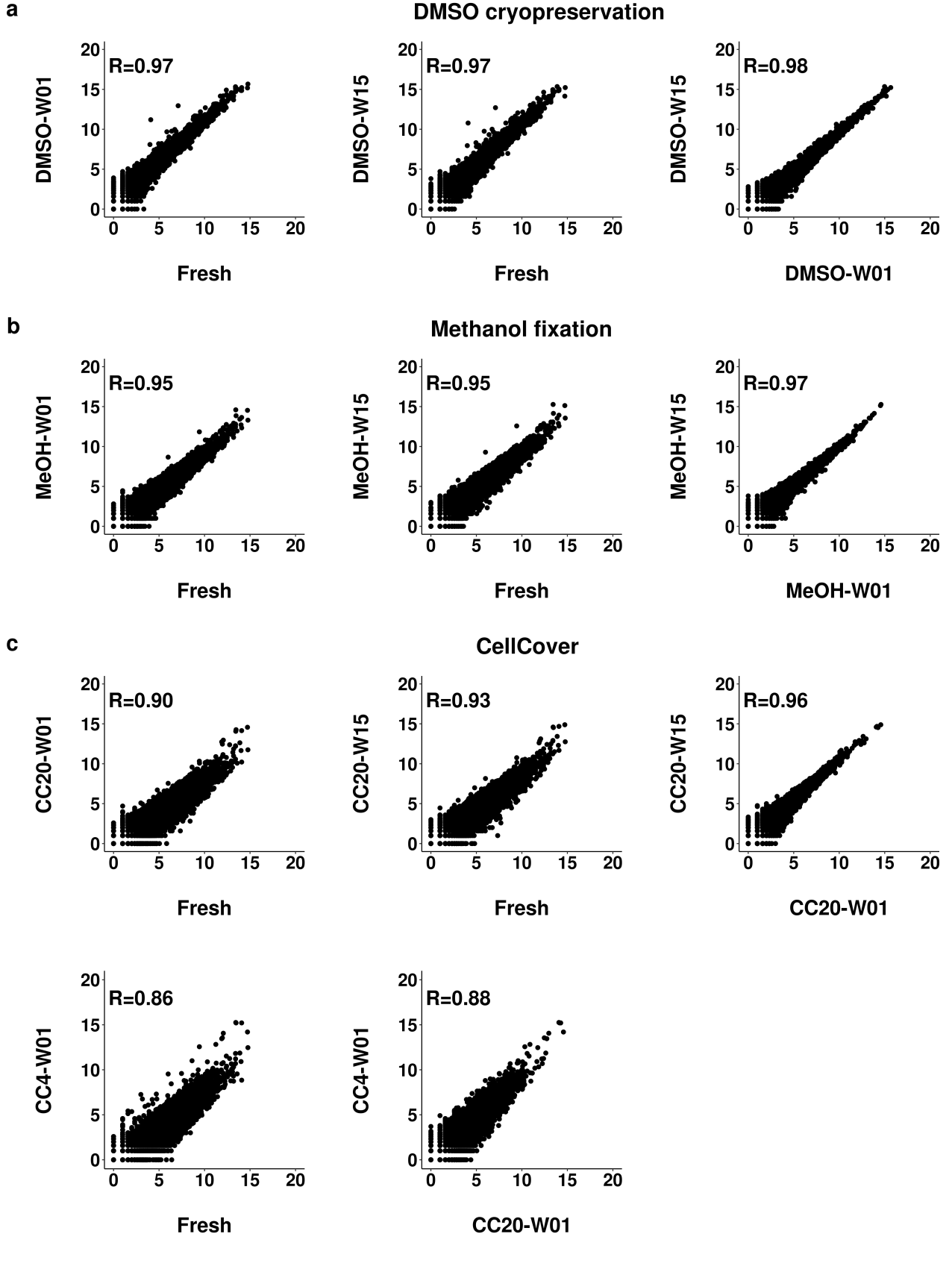


**Supplementary Figure S3. Pseudo-bulk gene expression correlation of fresh and preserved murine 3T3 cells.**

Scatter plots show the pairwise correlations of pseudo-bulk gene expression profiles from fresh cells and DMSO cryopreserved (DMSO) cells (a), methanol fixed (MeOH) cells (b) and cells preserved by CellCover reagent (c) at 4 °C (CC4) and -20 °C (CC20). Cells were stored for one week (W01) and 15 weeks (W15). Pearson correlation coefficient (R) indicates the degree of correlation. Data are shown for murine 3T3 cells from the species mixing experiment. Axes represent log_2_ (UMI+1) counts.

**Supplementary Figure S4.**

| **Sample** | **Fresh** | **DMSO cryopreservation** |
| --- | --- | --- |
| **NC1** | **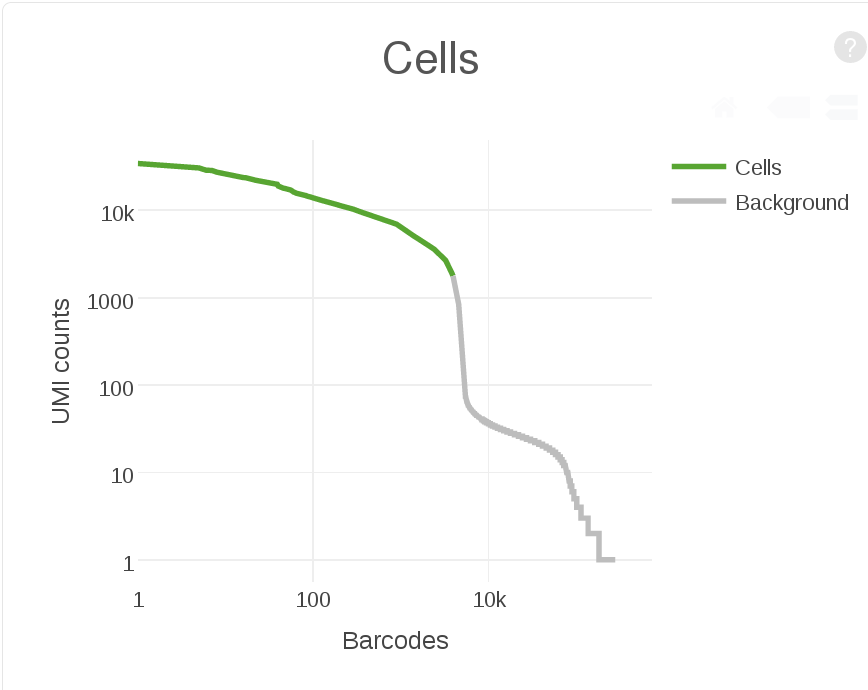** | **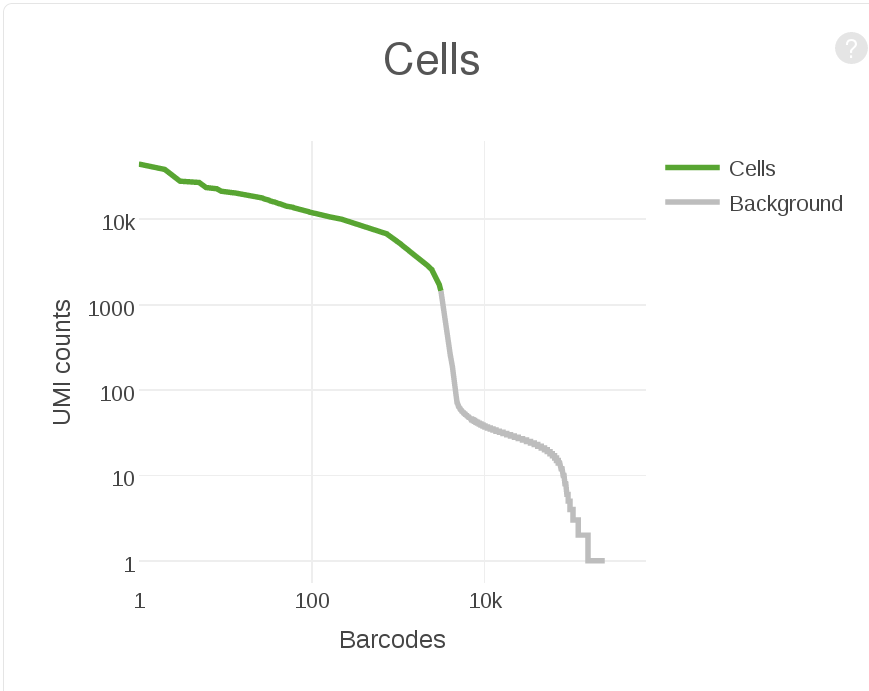** |
| **NC2** | **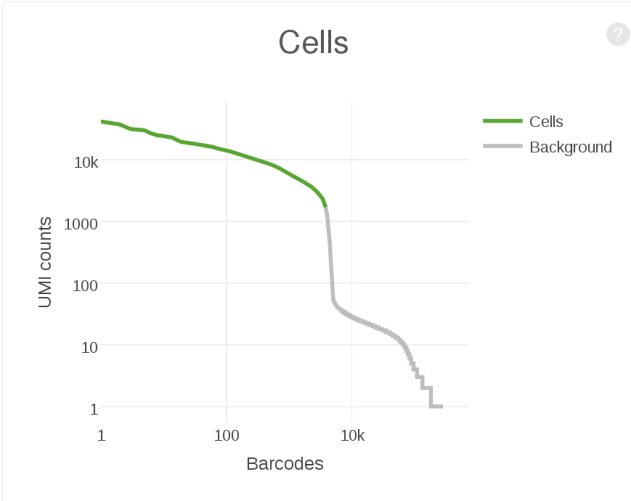** | **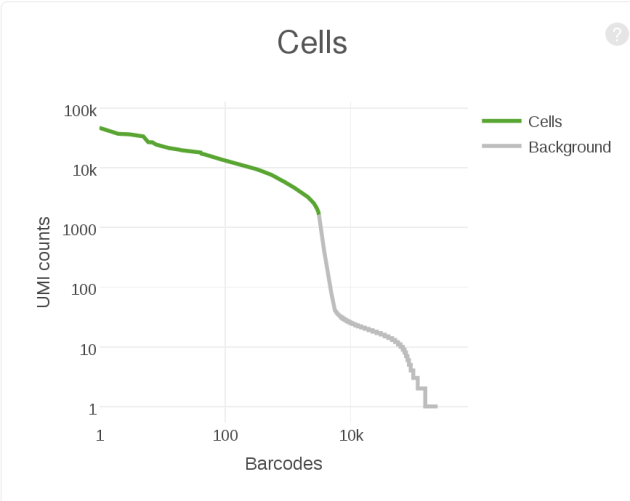** |
| **PC1** | **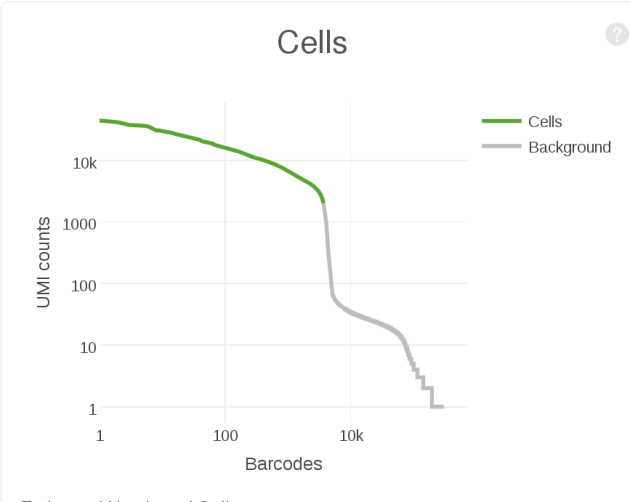** | **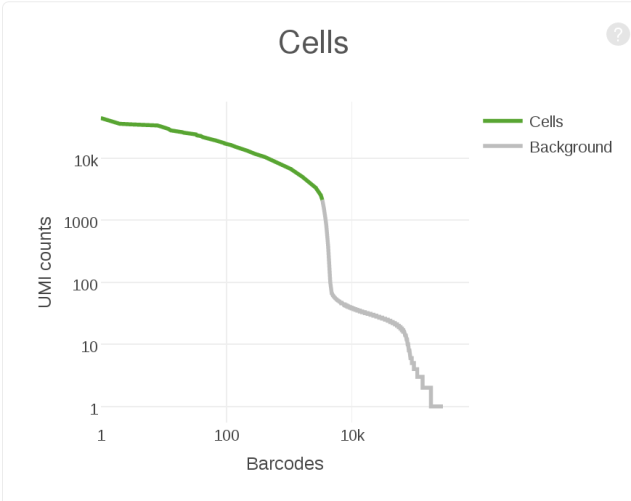** |
| **PC2** | **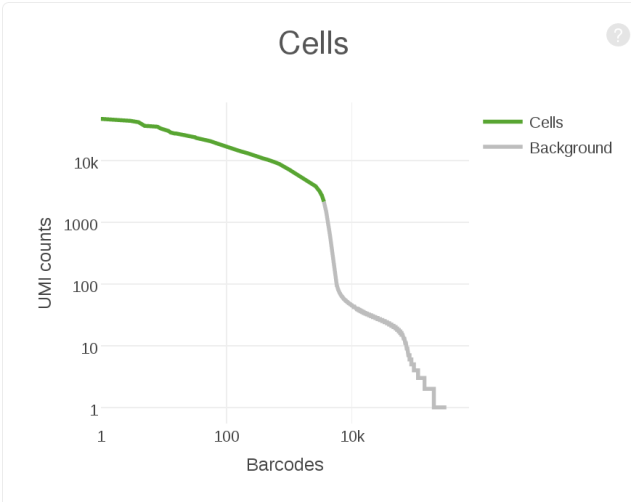** | **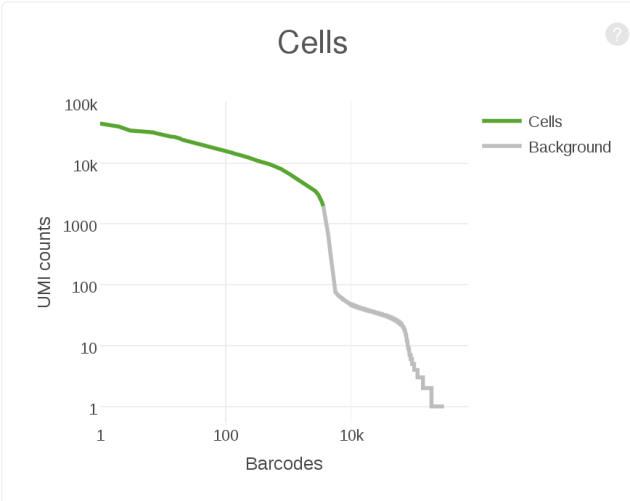** |

**Supplementary Figure S4. Barcode rank plots of fresh and preserved rat liver immune cells.**

The plots display the distribution of cell barcode counts and the corresponding total unique molecular identifier (UMI) counts of the fresh and DMSO cryopreserved cells as generated by the Cell Ranger pipeline. Data are shown for two negative (NC) and positive control (PC) animals, respectively. A steep drop-off of the curve indicates good separation of the cells (green) from background (grey), and therefore low ambient RNA contamination for both, fresh and preserved cells.

**Supplementary Figure S5.**


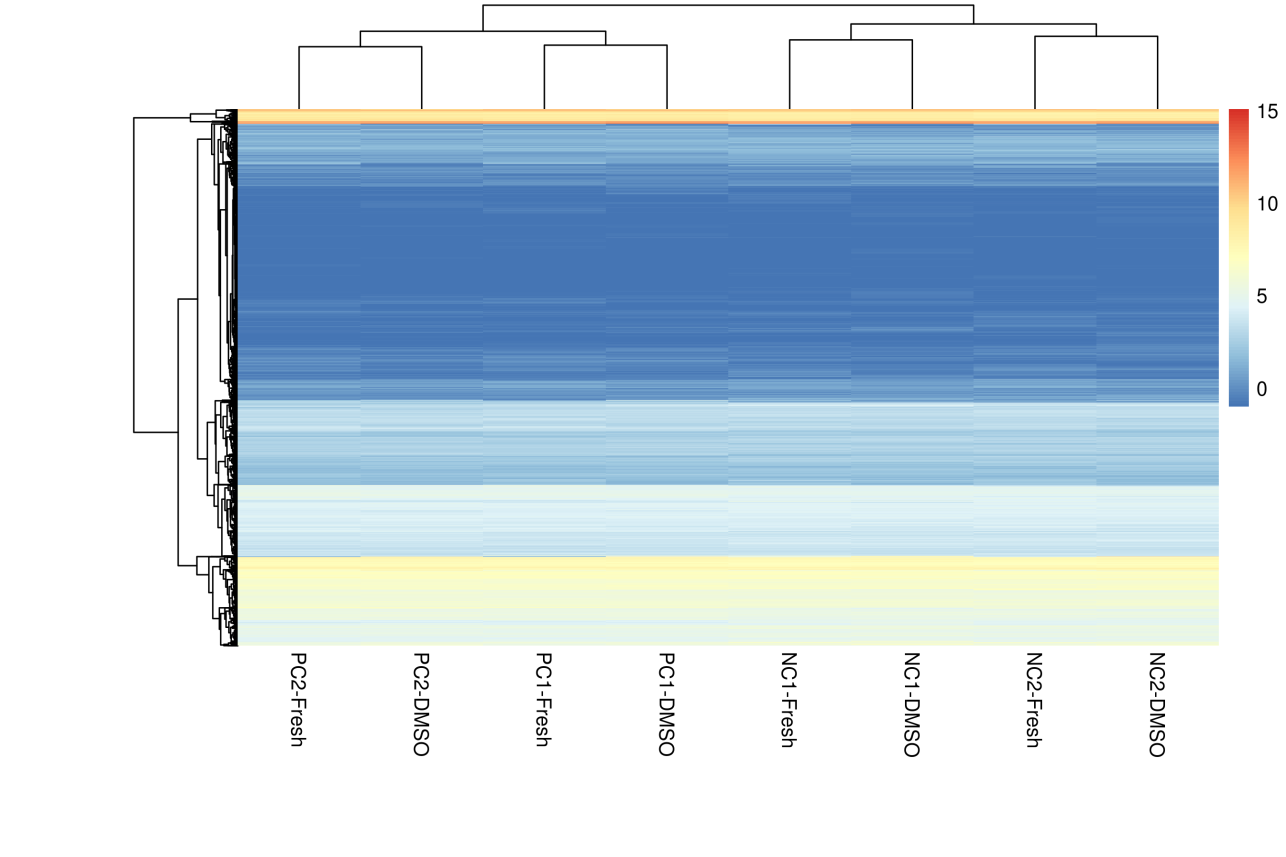


**Supplementary Figure S5. Hierarchical cluster analysis of fresh and preserved rat liver immune cells.**

Hierarchical cluster analysis based on the pseudo-bulk gene expression profiles of fresh and DMSO cryopreserved immune cells isolated from rat liver. Cells were isolated from two negative (NC) and positive control (PC) animals, respectively. Hierarchical clustering was performed on the entire gene set and color-coded gene expression levels are displayed in log_2_(counts per million).

**Supplementary Figure S6.**


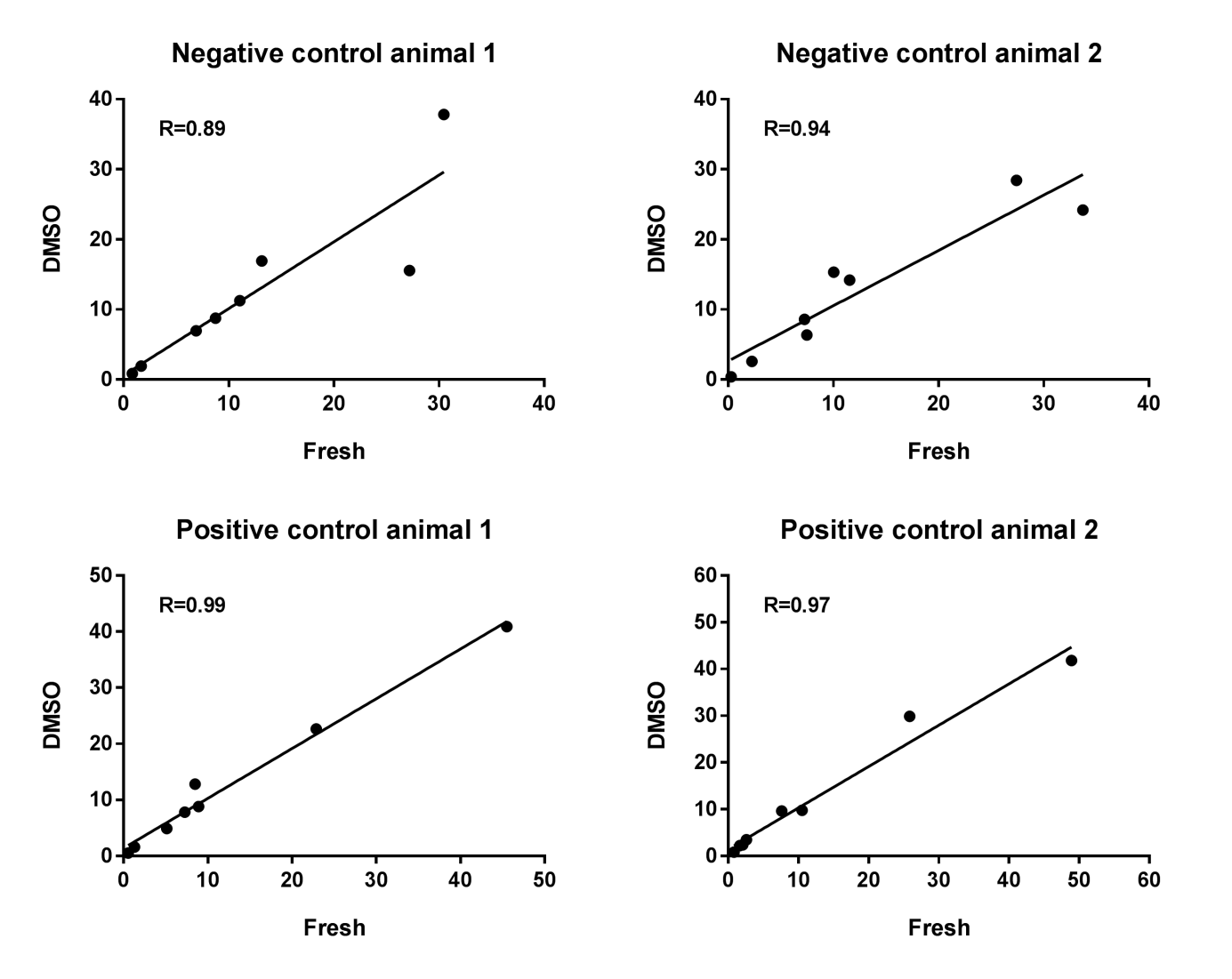


**Supplementary Figure S6. Correlation analysis of relative cell population abundance in fresh and cryopreserved immune cells.**

Eight major cell populations were identified in the primary immune cells isolated from rat liver. The relative abundance per cell population correlates strongly between fresh and DMSO cryopreserved samples per animal as shown in the scatter plots of negative control animal 1, negative control animal 2, positive control animal 1 and positive control animal 2. R, Pearson correlation coefficient.

**Supplementary Tables**

**Supplementary Table S1.**

Supplementary Table S1. Sample overview per single-cell RNA sequencing experiment.

| **Experiment** | **Protocol** | **Storage (weeks)** | **scRNA-seq platform** | **Estimated cell count** | **Mean reads per cell** |
| --- | --- | --- | --- | --- | --- |
| **Species mixing experiment** | -  DMSO cryopreservation  DMSO cryopreservation  Methanol fixation  Methanol fixation  CellCover reagent 4 °C  CellCover reagent -20 °C  CellCover reagent -20 °C | Fresh  1  15  1  15  1  1  15 | Drop-seq  Drop-seq  Drop-seq  Drop-seq  Drop-seq  Drop-seq  Drop-seq  Drop-seq | 1,100  1,553  1,292  606  953  1,029  603  1,322 | 18,650  14,846  14,745  28,557  15,244  14,789  14,144  6,075 |
| **Monocyte-derived macrophages** | -  DMSO cryopreservation  CellCover reagent -20 °C | Fresh  3  3 | Drop-seq  Drop-seq  Drop-seq | 451  571  127 | 76,226  63,786  190,542 |
| **Rat liver immune cells** | -  DMSO cryopreservation  -  DMSO cryopreservation  -  DMSO cryopreservation  -  DMSO cryopreservation | Fresh (NC1)  3 (NC1)  Fresh (NC2)  3 (NC2)  Fresh (PC1)  3 (PC1)  Fresh (PC2)  3 (PC2) | Chromium  Chromium  Chromium  Chromium  Chromium  Chromium  Chromium  Chromium | 4,719  3,603  4,336  3,542  4,121  3,897  4,333  4,325 | 48,845  43,453  49,853  46,329  51,631  50,147  50,837  44,402 |

Summary of the preservation protocol, storage period and single-cell RNA-seq platform used per sample as well as the estimated cell count per sample and the mean sequenced reads per cell. One replicate per condition was analysed for the Drop-seq experiments while cells of four rats (two positive (PC) and two negative control (NC) animals) were analysed on the Chromium system.

**Supplementary Table S2.**

Supplementary Table S2. Number of significantly altered genes between fresh and preserved species mixing samples.

| Protocol | Storage (weeks) | Species | Genes altered | Genes increased | | Genes decreased |
| --- | --- | --- | --- | --- | --- | --- |
| **DMSO cryopreservation** | 1  1  15  15 | Human  Mouse  Human  Mouse | 8  15  7  25 | | 4  14  6  19 | 4  1  1  6 |
| **Methanol fixation** | 1  1  15  15 | Human  Mouse  Human  Mouse | 36  25  82  55 | | 9  8  16  9 | 27  17  66  46 |
| **CellCover reagent 4 °C** | 1  1 | Human  Mouse | 813  400 | | 80  59 | 733  341 |
| **CellCover reagent -20 °C** | 1  1  15  15 | Human  Mouse  Human  Mouse | 486  244  311  135 | | 76  34  31  12 | 410  210  280  123 |

Number of genes that were at least 2-fold significantly (adj. *P* < 0.05) altered between fresh and preserved cells. Numbers are shown per preservation protocol and storage duration for human HEK293 and murine 3T3 cells, respectively.

**Supplementary Table S3.**

Supplementary Table S3. Number of significantly altered genes between fresh and preserved monocyte-derived macrophages.

| Protocol | Storage (weeks) | Species | Genes altered | Genes increased | | Genes decreased |
| --- | --- | --- | --- | --- | --- | --- |
| **DMSO cryopreservation** | 3 | Human | 6 | | 5 | 1 |
| **CellCover reagent -20 °C** | 3 | Human | 506 | | 99 | 407 |

Number of genes that were at least 2-fold significantly (adj. *P* < 0.05) altered between fresh and preserved human monocyte-derived macrophages. The capture of cDNA from methanol preserved cells and processing of cells stored in the CellCover reagent at 4 °C was not feasible, therefore data are not available for these preservation methods.

**Supplementary Table S4.**

Supplementary Table S4. Number of significantly altered genes between fresh and DMSO cryopreserved immune cells.

| Animal | Storage (weeks) | Species | Genes altered | Genes increased | | Genes decreased |
| --- | --- | --- | --- | --- | --- | --- |
| **Negative control 1** | 3 | Rat | 9 | | 6 | 3 |
| **Negative control 2** | 3 | Rat | 14 | | 10 | 4 |
| **Positive control 1** | 3 | Rat | 7 | | 5 | 2 |
| **Positive control 2** | 3 | Rat | 10 | | 9 | 1 |

Number of genes that were at least 2-fold significantly (adj. *P* < 0.05) altered between fresh and DMSO cryopreserved immune cells that were isolated from rat liver. Numbers are shown for both negative and positive control animals, respectively.

**Supplementary Methods**

**Generation of monocyte-derived macrophages**

Monocyte-derived macrophages were differentiated from isolated human monocytes. 200 mL of fresh blood were diluted with 200 mL of PBS/2 mM EDTA solution and 30 mL aliquots were transferred to Leucosep^TM^ tubes (Greiner Bio-One, Kremsmünster, Austria) filled with 15 mL of Ficoll^®^ Paque Plus (GE Healthcare, Chicago, IL). The tubes were centrifuged for 10 min at 1,000 g (breaks off) and peripheral blood mononuclear cells (PBMCs) were collected. PBMCs from two tubes were pooled, filled up to 50 mL with PBS/2 mM EDTA solution and centrifuged for 10 min at 250 g. Supernatants were discarded and the cell pellets of two tubes were resuspended, filled up to 50 mL with PBS/2 mM EDTA solution and centrifuged for 10 min at 200 g. Supernatants were again discarded and the cell pellets were resuspended in 10 ml of ACK Lysing Buffer (Gibco, Thermo Fisher Scientific, Waltham, MA). The cell suspensions were then incubated for 5 – 10 min at room temperature to lyse remaining erythrocytes. For the final wash step PBS/2 mM EDTA solution was added to a total volume of 50 mL and the tubes were centrifuged for 10 min at 250 g. PBMC pellets were finally pooled and resuspended in PBS. Monocytes were isolated from the purified PBMCs by the EasySep^TM^ Human Monocyte Isolation Kit (Stemcell Technologies, Vancouver, BC, Canada) following the manufacturer’s instructions.

In order to generate M1-like macrophages 3 x 10^6^ freshly isolated monocytes were cultivated per well of a 6-well Nunc UpCell^TM^ plate (Thermo Fisher Scientific, Waltham, MA) using X-VIVO^TM^ 10 Medium (Lonza, Basel, Switzerland) supplemented with 100 ng/ml of GM-CSF (rhGM-CSF, R&D Systems, Minneapolis, MN). Cells were incubated at 37 °C and 5 % CO_2_ throughout differentiation. On day 6 of the differentiation protocol 50 – 75 % of fresh medium supplemented with 100 ng/ml of GM-CSF was added per well. Monocyte-derived macrophages were harvested on day 11 of the differentiation protocol.
